# RNAi-Mediated Reverse Genetic Screen Identified *Drosophila* Chaperones Regulating Eye and Neuromuscular Junction Morphology

**DOI:** 10.1534/g3.117.041632

**Published:** 2017-05-08

**Authors:** Sandeep Raut, Bhagaban Mallik, Arpan Parichha, Valsakumar Amrutha, Chandan Sahi, Vimlesh Kumar

**Affiliations:** * Chaperone and Stress Biology Laboratory, Department of Biological Sciences, Indian Institute of Science Education and Research (IISER) Bhopal, Bhauri, Madhya Pradesh, India 462066; † Laboratory of Neurogenetics, Department of Biological Sciences, Indian Institute of Science Education and Research (IISER) Bhopal, Bhauri, Madhya Pradesh, India 462066

**Keywords:** *Drosophila*, chaperones, RNAi, eye morphogenesis, neuromuscular junction, Mutant Screen Report

## Abstract

Accumulation of toxic proteins in neurons has been linked with the onset of neurodegenerative diseases, which in many cases are characterized by altered neuronal function and synapse loss. Molecular chaperones help protein folding and the resolubilization of unfolded proteins, thereby reducing the protein aggregation stress. While most of the chaperones are expressed in neurons, their functional relevance remains largely unknown. Here, using bioinformatics analysis, we identified 95 *Drosophila* chaperones and classified them into seven different classes. Ubiquitous *actin5C*-Gal4-mediated RNAi knockdown revealed that ∼50% of the chaperones are essential in *Drosophila*. Knocking down these genes in eyes revealed that ∼30% of the essential chaperones are crucial for eye development. Using neuron-specific knockdown, immunocytochemistry, and robust behavioral assays, we identified a new set of chaperones that play critical roles in the regulation of *Drosophila* NMJ structural organization. Together, our data present the first classification and comprehensive analysis of *Drosophila* chaperones. Our screen identified a new set of chaperones that regulate eye and NMJ morphogenesis. The outcome of the screen reported here provides a useful resource for further elucidating the role of individual chaperones in *Drosophila* eye morphogenesis and synaptic development.

Within cells, proteins fold into three-dimensional conformations to attain their native state in order to achieve functionality. However, under physiological or environmental stress, proteins undergo misfolding that can lead to nonnative protein interactions and aggregation (Tyedmers *et al.* 2010). Cells have therefore evolved an intrinsic network of protein quality control machinery that functions to balance protein folding, misfolding, aggregation, and degradation; thereby maintaining protein homeostasis (proteostasis). This protein quality control machinery involves molecular chaperones that act as the first line of defense and participate in the refolding or, alternatively, the degradation of misfolded proteins (Kim *et al.* 2013).

Chaperones constitute diverse group of proteins that assist the noncovalent assembly/disassembly of other macromolecular structures (Liberek *et al.* 2008). Some, but not all, chaperones are also stress or heat shock proteins (HSPs) as their functional relevance increases under stress conditions, which otherwise may cause proteins to unfold and aggregate (Feder and Hofmann 1999). Chaperones are often classified according to their molecular weight, and members include Hsp100, Hsp90, Hsp70, Hsp60, Hsp40 (DnaJ), prefoldins, and the small HSPs (sHSPs) (Gong *et al.* 2009). They regulate multiple aspects of cellular physiology. For instance, in addition to their fundamental roles in *de novo* protein folding, chaperones also regulate critical cellular processes such as exocytosis and endocytosis (Young *et al.* 2003), autophagy (Kaushik and Cuervo 2012), apoptosis (Soti *et al.* 2003), and proteasomal degradation (Hohfeld *et al.* 2001).

Postmitotic cells like neurons are particularly prone to detrimental effects of misfolded/aggregated proteins as they cannot dilute toxic protein aggregates by cell division, which may result in an accumulation of misfolded proteins (Muchowski and Wacker 2005). The disruption of neuronal proteostasis may lead to aberrantly folded proteins that typically lose their functions. The accumulation of misfolded and aggregated proteins is also cytotoxic and has been implicated in the pathogenesis of many neurodegenerative diseases (Ross and Poirier 2004).

It is widely appreciated that, when compromised, chaperone activity and proteasomal machinery fall short of compensating for the protein damage caused by misfolding and free radicals. Moreover, this leads to the accumulation of protein aggregates, a situation called “chaperone overload” (Soti *et al.* 2003). Studies in different organisms have reported a tight correlation between age-dependent protein misfolding and weakened proteasomal activity in neurons, which makes them vulnerable to protein aggregation (Morimoto 2008). Elevated heat shock response promotes longevity (Yokoyama *et al.* 2002; Vos *et al.* 2016; Morley and Morimoto 2004) whereas defective chaperone molecules along with proteotoxic stress result in early aging (Macario and Conway de Macario 2002).

HSPs have been reported to play a key role in neurogenesis (Calabrese *et al.* 2002) and are constitutively expressed in neurons (Brown and Rush 1990). Most HSPs show higher expression levels in neuronal tissues (D’Souza and Brown 1998). Interestingly, several reports suggest that a compromised chaperone activity (*e.g.*, for Hsp40 and Hsp70) leads to impaired neurotransmission signifying their neuronal function (Bronk *et al.* 2001; Morgan *et al.* 2001). Chaperones are induced in various neuropathies, which points toward their neuroprotective role (Yenari *et al.* 1999). While chaperones are widely expressed in neurons, very little is known about their specific roles in the nervous system (Muchowski 2002).

In order to determine their functional relevance *in vivo*, we performed an RNAi-mediated targeted genetic screen for all *Drosophila* chaperone proteins identified using bioinformatics analysis. We identified several novel chaperones for which neither gene group membership nor protein family has been assigned in FlyBase. We classified *Drosophila* chaperones into seven different classes based on their domain organization. Next, we ubiquitously knocked-down 167 RNAi lines for a total of 95 *Drosophila* chaperones using *actin5C*-Gal4 and selected essential chaperones. To better understand the cellular functions of essential chaperones, we knocked-down these genes in eyes and identified several chaperones involved in eye morphogenesis and/or rhabdomere development. Finally, neuron-specific knockdown of RNAi lines corresponding to 42 essential chaperones identified several candidates that altered *Drosophila* NMJ development. We shortlisted nine candidates belonging to different chaperone families based on the severity of NMJ structural defects. Neuronal knockdown of some of these candidates resulted in a larval crawling defect as well as compromised adult climbing ability. We suggest that further analysis of the individual chaperones identified in this screen would help us better understand the molecular mechanisms and pathways that they regulate during *Drosophila* eye and NMJ morphogenesis.

## Materials and Methods

### In silico identification of Drosophila chaperones

Seven protein families (sHsps, Hsp40, prefoldins, Hsp60/Cpn60, Hsp70, Hsp90, and Hsp100) were considered as canonical chaperone families based on thorough literature analysis (Hartl and Hayer-Hartl 2002; Gong *et al.* 2009; Saibil 2013). The *Saccharomyces cerevisiae* “chaperome” is well characterized with the exact number of candidate proteins belonging to each chaperone family known, and thus the same was used as a template to identify the *Drosophila* “chaperome.” BLASTP searches were performed in FlyBase (http://flybase.org/blast/) and PSI-BLAST searches were conducted in the NCBI database targeted to *Drosophila melanogaster* (https://blast.ncbi.nlm.nih.gov/Blast.cgi) using the protein sequence of each *S. cerevisiae* chaperone as a query. From the results of BLAST searches, nonredundant hits were listed. Each protein from the list was analyzed for domain organization using the SMART database (http://smart.embl-heidelberg.de/) (Schultz *et al.* 1998). Based on the domain organization and information available in FlyBase, *Drosophila* chaperones were classified into seven chaperone families (*e.g.*, J-domain-containing chaperones were enlisted as members of the Hsp40 family) to make a comprehensive list of *Drosophila* chaperones. The chaperones for which neither gene group membership nor protein family was assigned in FlyBase were considered to be novel chaperones. Results were compared with the Heat Shock Protein Information Resource HSPIR database (http://pdslab.biochem.iisc.ernet.in/hspir/) (Ratheesh Kumar *et al.* 2012).

### Fly strains and genetics

RNAi lines used in this study were procured from the Vienna *Drosophila* Resource Centre (VDRC) (Dietzl *et al.* 2007). Flies were cultured at 25° on a corn meal-agar medium containing yeast granules. A bipartite UAS/Gal4 system (Brand and Perrimon 1993) was used for tissue-specific knockdown of chaperones. The Gal4 driver lines used in this study were *actin5C*-Gal4 (BDSC-25374), *ey*-Gal4 (BDSC-5534), *D42*-Gal4 (BDSC-8816), and *elav^C155^*-Gal4 (BDSC-458). The white-eyed *w^1118^ Drosophila* strain was used as control except where indicated. All the RNAi knockdown experiments were performed at 29° under controlled humidity (60% RH) in an incubator. To screen for essential chaperones, *actin5C*-Gal4 without *Dicer-2* was used. While *Dicer-2* does enhance the efficiency of knockdown, it may also cause off-target knockdowns and lead to additional lethality (Dietzl *et al.* 2007). Hence, we chose not to use *Dicer-2* for screening chaperones.

### Bright-field imaging

Seven-day-old flies were anesthetized using ether and photomicrographs of eyes were taken using a color camera mounted on a Leica M205FA Stereo Zoom Microscope. All images were processed using Adobe Photoshop 7.0 (Adobe Systems, San Jose, CA).

### Antibodies and immunocytochemistry

Wandering third instar larvae were dissected on a sylgard dish in cold calcium-free HL3 saline (70 mM NaCl, 5 mM KCl, 20 mM MgCl_2_, 10 mM NaHCO_3_, 5 mM Trehalose, 115 mM sucrose, 5 mM HEPES, and 5 mM EGTA) and fixed in 4% paraformaldehyde in PBS for 30 min. Larval fillets were then washed in PBS containing 0.2% Triton X-100, blocked for 1 hr in 5% normal goat serum, and then incubated overnight at 4° with the primary antibody. Monoclonal antibodies (anti-CSP and mAb22C10) were obtained from the Developmental Studies Hybridoma Bank (University of Iowa) and were used at 1:50 dilution. Fluorophore-coupled secondary antibodies Alexa Fluor 488, Alexa Fluor 568, or Alexa Fluor 633 (Molecular Probes and Thermo Fisher Scientific) were used at 1:800 dilution. Alexa 488-conjugated anti-HRP was used at 1:800 dilution. Stained larval preparations were mounted in VECTASHIELD (Vector Laboratories) and imaged with a laser scanning confocal microscope (LSM 780; Carl Zeiss). All images were processed with Adobe Photoshop 7.0 (Adobe Systems).

### Futsch loop quantification

Third instar larval fillets were double stained with HRP and mAb22C10. Confocal (LSM 780; Carl Zeiss) images of NMJ at muscle 6/7, A2 hemisegment were captured using a 63 × /1.4 NA objective. Images were digitally magnified using ImageJ (NIH) and the total number of boutons was first determined by manually counting the number of HRP-positive varicosities. This was followed by counting the number of complete looped structures that colocalized with HRP. Incomplete loops and loops with diffused or interrupted staining were not included in the count. The total number of loops was divided by the total bouton number to arrive at the percentage of boutons with complete Futsch positive loops (Roos *et al.* 2000; Hummel *et al.* 2000).

### Locomotive behavioral assays

The third instar larval crawling and Rapid Iterative Negative Geotaxis assays with adult flies were performed as previously described (Nichols *et al.* 2012; Gargano *et al.* 2005). *elav^C155^*-Gal4/+ was used as control for these assays upon pan-neuronal knockdown and *D42*-Gal4/+ was used as control for behavioral assays upon motor neuron-specific knockdown. A Nikon COOLPIX P600 camera was used for video recording and imaging. Statistical analysis was performed using GraphPad Prism software (GraphPad Software, San Diego).

#### Larval crawling assay:

Each vial harboring third instar larvae had 10 ml of 20% sucrose solution poured in and was left for 15 min to let the larvae float on top. Third instar larvae were gently collected using a 1.0 ml pipette with a cut tip and washed twice with deionized water. Ten larvae of each genotype were subsequently transferred to a 2% agarose gel in a Petri dish with gridline markings spaced at 0.5 cm. The larvae were allowed to acclimatize to the new environment before videotaping. The average distance crawled (in centimeters) by larvae was calculated based on the average number of gridlines passed by the posterior ends of the larvae in 30 sec. All statistical analysis conducted is based on one-way ANOVA with a *post hoc* Tukey’s test for multiple comparisons.

#### Climbing ability test:

Five-day-old flies of respective genotypes were collected in transparent 50 ml falcon tubes marked with a medial line. All the tubes were arranged in a holder and a camera was set at a 1 meter distance from the Falcon holder. Flies were allowed to settle at the bottom by gently tapping the Falcon holder thrice on the surface. Negative geotaxis of the flies was videotaped and the number of flies crossing the medial line in 5 sec was counted for each genotype. The assay was repeated 10 times, and the climbing ability was presented as the average percentage of flies crossing medial line in 5 sec. All the statistical analysis conducted is based on one-way ANOVA with a *post hoc* Tukey’s test for multiple comparisons.

### Data availability

All supportive data and materials generated in this study will be made available upon request. Data supporting total bouton number and Futsch loop quantification are provided as Supplemental Material, Table S3.

## Results

### Identification and classification of Drosophila chaperones

Based on the published literature, sHsps, prefoldins, Hsp40, Hsp60, Hsp70, Hsp90, and Hsp100 are considered as canonical chaperone families. Members of these chaperone families interact with each other to form a complex network called as the “chaperome,” which monitors cellular proteostasis (Brehme *et al.* 2014). These chaperones are well characterized in *S. cerevisiae* and have been classified into different families (Gong *et al.* 2009). Thus, BLASTP searches were performed using sequences of *S. cerevisiae* chaperones to identify *Drosophila* counterparts, which were further classified into different chaperone families based on their domain organization. We report the most comprehensive list of *Drosophila* chaperones to date, with all details including their FlyBase ID, symbol, gene name, alternate name, gene group membership, and protein family (Table 1). The total number of chaperones in *Drosophila* is higher when compared with *S cerevisiae*. While 63 chaperones belonging to seven classes have been reported for *S. cerevisiae* (Gong *et al.* 2009), our analysis in *Drosophila* revealed a total of 95 chaperones, which we classified into seven families. Interestingly, Hsp40 outnumbers other classes of chaperones, as evident from our bioinformatics analysis (Table 1). Moreover, we identified seven novel chaperones (CG15676, CG15266, CG2911, CG1416, CG6355, CG7182, and CG4538) for which information regarding gene group membership and protein family has not been assigned in FlyBase (Table 1).

**Table 1:** Identification and classification of *Drosophila* chaperones

Sr. No.	Annotation	FlyBase ID	Gene Name	Symbol	Alternate Name	Gene Group Membership	Protein Family
Small heat shock proteins							
1	CG4167	FBgn0001227	Heat shock gene 67Ba	Hsp67Ba	gene 1, gene1	Small heat shock proteins	Small heat shock protein (HSP20) family
2	CG4183	FBgn0001225	Heat shock protein 26	Hsp26	DmHsp26, hsp26, 26	Small heat shock proteins	Small heat shock protein (HSP20) family
3	CG4190	FBgn0001229	Heat shock gene 67Bc	Hsp67Bc	gene 3	Small heat shock proteins	Small heat shock protein (HSP20) family
4	CG4460	FBgn0001223	Heat shock protein 22	Hsp22	DmHsp22, CG32041	Small heat shock proteins	Small heat shock protein (HSP20) family
5	CG4461	FBgn0035982		CG4461	Hsp20	Small heat shock proteins	
6	CG4463	FBgn0001224	Heat shock protein 23	Hsp23	DmHsp23, 23	Small heat shock proteins	Small heat shock protein (HSP20) family
7	CG4466	FBgn0001226	Heat shock protein 27	Hsp27	Hsp28, DmHsp27, Dhsp27, hsp 27	Small heat shock proteins	Small heat shock protein (HSP20) family
8	CG4533	FBgn0011296	Lethal (2) essential for life	l(2)efl	Cryab	Small heat shock proteins	Small heat shock protein (HSP20) family
9	CG7409	FBgn0035817		CG7409		Small heat shock proteins	
10	CG13133	FBgn0032181		CG13133		Small heat shock proteins	
11	CG14207	FBgn0031037		CG14207		Small heat shock proteins	
Prefoldins							
1	CG6302	FBgn0010741	Prefoldin 2	Pfdn2	l(3)01239		Prefoldin subunit β family
2	CG6719	FBgn0264694	Merry-go-round	Mgr			Prefoldin subunit α family
3	CG7048	FBgn0038976	Prefoldin 5	Pfdn5			Prefoldin subunit α family
4	CG7770	FBgn0036918	Prefoldin 6	Pfdn6			Prefoldin subunit β family
5	CG10635	FBgn0035603	Prefoldin 4	Pfdn4			Prefoldin subunit β family
6	CG15266	FBgn0259982	Lethal (2) 35Cc	l(2)35Cc			
7	CG15676	FBgn0034651		CG15676			
Heat shock protein 40 kDa (HSP40)							
1	CG1107	FBgn0037218	Auxilin	aux	dAux	Heat shock protein 40/DnaJ cochaperones	
2	CG1409	FBgn0029964		CG1409		Heat shock protein 40/DnaJ cochaperones	
3	CG1416	FBgn0032961		CG1416			
4	CG2239	FBgn0027654	jdp	jdp	dJDP	Heat shock protein 40/DnaJ cochaperones	
5	CG2790	FBgn0027599		CG2790		Heat shock protein 40/DnaJ cochaperones	
6	CG2887	FBgn0030207		CG2887		Heat shock protein 40/DnaJ cochaperones	
7	CG2911	FBgn0037350		CG2911			
8	CG3061	FBgn0038195		CG3061		Heat shock protein 40/DnaJ cochaperones	
9	CG4164	FBgn0031256	Shriveled	shv		Heat shock protein 40/DnaJ cochaperones	
10	CG4599	FBgn0032586	Tetratricopeptide repeat protein 2	Tpr2	dTPR2	Heat shock protein 40/DnaJ cochaperones	
11	CG5001	FBgn0031322		CG5001		Heat shock protein 40/DnaJ cochaperones	
12	CG5268	FBgn0038387	Black pearl	Blp	l(3)01618	Heat shock protein 40/DnaJ cochaperones	Belongs to the TIM16/PAM16 family
13	CG5504	FBgn0002174	Lethal (2) tumorous imaginal discs	l(2)tid		Heat shock protein 40/DnaJ cochaperones	
14	CG6395	FBgn0004179	Cysteine string protein	Csp	Dcsp, ab49	Heat shock protein 40/DnaJ cochaperones	
15	CG6693	FBgn0037878		CG6693		Heat shock protein 40/DnaJ cochaperones	
16	CG7130	FBgn0037151		CG7130		Heat shock protein 40/DnaJ cochaperones	
17	CG7133	FBgn0037150		CG7133		Heat shock protein 40/DnaJ cochaperones	
18	CG7387	FBgn0035852		CG7387		Heat shock protein 40/DnaJ cochaperones	
19	CG7394	FBgn0036173		CG7394		Heat shock protein 40/DnaJ cochaperones	Belongs to the TIM14 family
20	CG7556	FBgn0030990		CG7556		Heat shock protein 40/DnaJ cochaperones	
21	CG7872	FBgn0030658		CG7872		Heat shock protein 40/DnaJ cochaperones	Belongs to the DNAJC25 family
22	CG8014	FBgn0015477	Receptor-mediated endocytosis 8	Rme-8	l(2)45Ba	Heat shock protein 40/DnaJ cochaperones	
23	CG8286	FBgn0037718	P58IPK	P58IPK		Heat shock protein 40/DnaJ cochaperones	
24	CG8448	FBgn0034091	Mrj	Mrj	dMRJ	Heat shock protein 40/DnaJ cochaperones	
25	CG8476	FBgn0038127		CG8476		Heat shock protein 40/DnaJ cochaperones	
26	CG8531	FBgn0033918		CG8531		Heat shock protein 40/DnaJ cochaperones	
27	CG8583	FBgn0035771	Secretory 63	Sec63		Heat shock protein 40/DnaJ cochaperones	
28	CG8863	FBgn0038145	DnaJ-like-2	Droj2		Heat shock protein 40/DnaJ cochaperones	
29	CG9089	FBgn0030805	Wurst	Wus		Heat shock protein 40/DnaJ cochaperones	
30	CG9828	FBgn0032474	DnaJ homolog	DnaJ-H		Heat shock protein 40/DnaJ cochaperones	
31	CG10375	FBgn0039116		CG10375		Heat shock protein 40/DnaJ cochaperones	
32	CG10565	FBgn0037051		CG10565		Heat shock protein 40/DnaJ cochaperones	
33	CG10578	FBgn0263106	DnaJ-like-1	DnaJ-1	Hsp40, dHdj1, DnaJ1, droj1	Heat shock protein 40/DnaJ cochaperones	
34	CG11035	FBgn0037544		CG11035		Heat shock protein 40/DnaJ cochaperones	
35	CG12020	FBgn0035273		CG12020		Heat shock protein 40/DnaJ cochaperones	
36	CG14650	FBgn0037252		CG14650		Heat shock protein 40/DnaJ cochaperones	
37	CG17187	FBgn0037882		CG17187		Heat shock protein 40/DnaJ cochaperones	
38	CG30156	FBgn0050156		CG30156		Heat shock protein 40/DnaJ cochaperones	
39	CG32640	FBgn0052640		CG32640		Heat shock protein 40/DnaJ cochaperones	
40	CG32641	FBgn0052641		CG32641		Heat shock protein 40/DnaJ cochaperones	
41	CG32727	FBgn0265265		CG32727		Heat shock protein 40/DnaJ cochaperones	
42	CG34246	FBgn0263606	Heat shock protein cognate 20	Hsc20	l(3)72Do	Heat shock protein 40/DnaJ cochaperones	
43	CG40178	FBgn0058178		CG40178		Heat shock protein 40/DnaJ cochaperones	
44	CG42567	FBgn0260775	DnaJ-like-60	DnaJ-60	DnaJ60	Heat shock protein 40/DnaJ cochaperones	
45	CG43322	FBgn0263027		CG43322		Heat shock protein 40/DnaJ cochaperones	
Heat shock protein 60 kDa (HSP60)							
1	CG2830	FBgn0011244	Heat shock protein 60B	Hsp60B	Hsp64	Heat shock protein 60 chaperonins group i	Chaperonin (HSP60) family
2	CG5374	FBgn0003676	Chaperonin-containing TCP1 subunit 1	CCT1	T-cp1, T-cpl, tcp1, Tcp1-like, Tcp1-α	Heat shock protein 60 chaperonins group ii	TCP-1 chaperonin family
3	CG5525	FBgn0032444	Chaperonin-containing TCP1 subunit 4	CCT4	CCT4, Tcp1-δ	Heat shock protein 60 chaperonins group ii	
4	CG6355	FBgn0028741	Fab1 kinase	fab1			
5	CG7033	FBgn0030086	Chaperonin-containing TCP1 subunit 2	CCT2	CCT2, Tcp1-β	Heat shock protein 60 chaperonins group ii	
6	CG7235	FBgn0031728	Heat shock protein 60C	Hsp60C	Hsp64	Heat shock protein 60 chaperonins group i	Chaperonin (HSP60) family
7	CG8231	FBgn0027329	Chaperonin-containing TCP1 subunit 6	CCT6	Tcp-1ζ, TCP-1ζ, l(1)G0022	Heat shock protein 60 chaperonins group ii	
8	CG8258	FBgn0284436	Chaperonin-containing TCP1 subunit 8	CCT8	Tcp1-θ	Heat shock protein 60 chaperonins group ii	
9	CG8351	FBgn0037632	Chaperonin-containing TCP1 subunit 7	CCT7	tcp-1η, Cct7	Heat shock protein 60 chaperonins group ii	
10	CG8439	FBgn0010621	Chaperonin-containing TCP1 subunit 5	CCT5	cct5, Tcp1-ɛ	Heat shock protein 60 chaperonins group ii	
11	CG8977	FBgn0015019	Chaperonin-containing TCP1 subunit 3	CCT3	Cctγ, Y, Cctg, cct-γ, TCPG_DROME, Tcp1-γ, Cctγ	Heat shock protein 60 chaperonins group ii	TCP-1 chaperonin family
12	CG12101	FBgn0015245	Heat shock protein 60A	Hsp60A	hsp60, l(1)BP5, Hsp60A, Dmhsp60, 12, Hsp64	Heat shock protein 60 chaperonins group i	Chaperonin (HSP60) family
13	CG16954	FBgn0032525	Heat shock protein 60D	Hsp60D		Heat shock protein 60 chaperonins group i	
Heat Shock Protein 70 kDa (HSP70)							
1	CG2918	FBgn0023529		CG2918	EG:25E8.1, GRP170	Atypical heat shock protein 70 chaperones	
2	CG4147	FBgn0001218	Heat shock 70-kDa protein cognate 3	Hsc70-3	Bip, HSC3, Hsc70, Grp78, Hsc-70-3, dBiP	Heat shock protein 70 chaperones	Heat shock protein 70 family
3	CG4264	FBgn0266599	Heat shock protein cognate 4	Hsc70-4	Hsc4, Hsc70, hsp70	Heat shock protein 70 chaperones	Heat shock protein 70 family
4	CG5436	FBgn0001230	Heat shock protein 68	Hsp68	68	Heat shock protein 70 chaperones	Heat shock protein 70 family
5	CG5834	FBgn0051354	Heat shock protein 70Bbb	Hsp70Bbb	Hsp70, Hsp70B	Heat shock protein 70 chaperones	Heat shock protein 70 family
6	CG6489	FBgn0013279	Heat shock protein 70Bc	Hsp70Bc	Hsp70, Hsp70B, hsp-70, dhsp70	Heat shock protein 70 chaperones	Heat shock protein 70 family
7	CG6603	FBgn0026418	Hsc70Cb	Hsc70Cb	HSC70	Atypical heat shock protein 70 chaperones	
8	CG7182	FBgn0035878		CG7182			
9	CG7756	FBgn0001217	Heat shock protein cognate 2	Hsc70-2	Hsc70, HSC2	Heat shock protein 70 chaperones	Heat shock protein 70 family
10	CG8542	FBgn0001220	Heat shock protein cognate 5	Hsc70-5	Hsc70, Hsc5	Heat shock protein 70 chaperones	Heat shock protein 70 family
11	CG8937	FBgn0001216	Heat shock protein cognate 1	Hsc70-1	Hsc70	Heat shock protein 70 chaperones	Heat shock protein 70 family
12	CG18743	FBgn0013276	Heat shock protein 70Ab	Hsp70Ab	Hsp70, Hsp70A, hsp-70, dhsp70	Heat shock protein 70 chaperones	Heat shock protein 70 family
13	CG31359	FBgn0013278	Heat shock protein 70Bb	Hsp70Bb	Hsp70, Hsp70B, hsp-70, dhsp70	Heat shock protein 70 chaperones	Heat shock protein 70 family
14	CG31366	FBgn0013275	Heat shock protein 70Aa	Hsp70Aa	Hsp70, Hsp70A, hsp-70, dhsp70	Heat shock protein 70 chaperones	Heat shock protein 70 family
15	CG31449	FBgn0013277	Heat shock protein 70Ba	Hsp70Ba	Hsp70, Hsp70B, hsp-70, dhsp70	Heat shock protein 70 chaperones	Heat shock protein 70 family
Heat shock protein 90 kDa (HSP90)							
2	CG1242	FBgn0001233	Heat shock protein 83	Hsp83	Hsp90, Hsp82, E(sina)2, 83	Heat shock protein 90 chaperones	Heat shock protein 90 family
1	CG3152	FBgn0026761	Trap1	Trap1		Heat shock protein 90 chaperones	
3	CG5520	FBgn0039562	Glycoprotein 93	Gp93		Heat shock protein 90 chaperones	
Heat shock protein 100 kDa (HSP100)							
1	CG4538	FBgn0038745		CG4538			

The comprehensive list of all *Drosophila* chaperones is obtained by using bioinformatics analysis. BLASTP searches in FlyBase (http://flybase.org/) and PSI-BLAST searches in NCBI using protein sequences of yeast chaperones as query sequences were performed. A comprehensive list from nonredundant hits of each BLASTP search was further analyzed for domain organization and the validated candidates are reported as a member of respective chaperone families in *Drosophila* based on their domain organization. In total, *Drosophila* genome contains 95 chaperones which we classify into seven families. The list of chaperones belonging to each class is shown in the table. There are several chaperones in our comprehensive list for which protein family or gene group membership has not been assigned in FlyBase. The chaperones with no data on gene family and gene group membership are considered as novel chaperones and highlighted in the table. Sr., serial number; No., number; ID, identifier.

### Ubiquitous knockdown identified several essential chaperones in Drosophila

**Figure 1:**
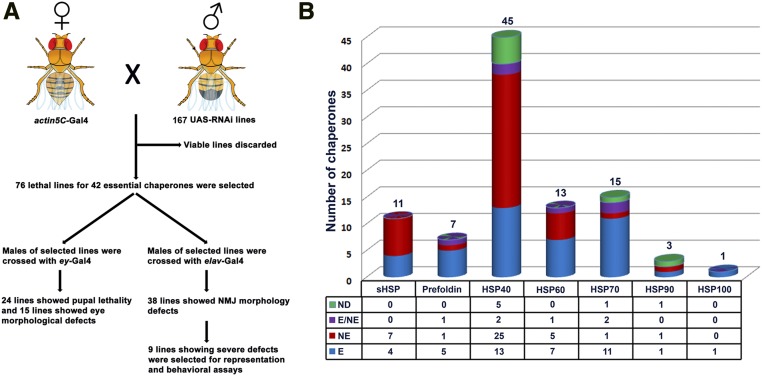
Workflow for the RNAi screen and list of *Drosophila* chaperones. (A) Schematic representation of workflow for UAS-RNAi for identification of their neuronal function in *Drosophila*. A total of 167 RNAi lines corresponding to 95 chaperones were crossed with *actin5C*-Gal4 for ubiquitous knockdown. The candidate chaperones responsible for lethal events in the F1 generation were considered as essential. All the essential line were then crossed with either *ey*-Gal4 (to identify chaperones required in eye morphogenesis) or with pan-neuronal *elav^C155^*-Gal4 (to identify chaperones required for neuronal function for which NMJ morphology was used as readout). Detailed analysis is shown in Table S1 and Table S2. (B) Histogram showing seven families of chaperones in *Drosophila*. The number above the histogram represents the total number of chaperones in each family. The Hsp40 family dominated the list, while only one Hsp100 was identified in *Drosophila*. The table below the histograms shows the number of essential chaperones (E), nonessential chaperones (NE), chaperones for which 50% of lines were lethal (E/NE), and genes for which lines could not be procured (ND). Detailed analysis of *actin5C*- Gal4 mediated knockdown is shown in Table S1. HSP, heat shock protein; NMJ, neuromuscular junction; RNAi, RNA interference; sHSP, small HSP; UAS, upstream activation sequence.

The RNAi technique has evolved to be a powerful approach to perform genome-wide or targeted reverse genetic screens to identify genes involved in various cellular pathways (Sharma and Rao 2009; Mohr 2014; Chen and Xu 2016; Agrawal and Hardin 2016; Vissers *et al.* 2016). For instance, such screens have identified novel genes involved in *Drosophila* nervous system development (Koizumi *et al.* 2007) and wound closure (Lesch *et al.* 2010). While most of the chaperones are expressed in the nervous system, their functional requirements in neurons remain largely unknown. To address this, we performed an RNAi screen to identify *Drosophila* chaperones with neuron-specific functions. We first identified the essential chaperones in *Drosophila* by *actin5C*-Gal4-mediated knockdown of 168 RNAi lines against 95 chaperone genes. Lines that did not produce viable F1 progeny were considered lethal. Since RNAi-mediated knockdown may cause off-target effects or inefficient knockdown (Dietzl *et al.* 2007), we used multiple RNAi lines for each chaperone gene subject to its availability at the VDRC. Chaperones for which the proportion of lethal lines was more than the viable lines were considered as essential chaperones (Figure 1). Due to this conservative approach, our list of 42 essential chaperones may be an underestimate in *Drosophila*. Alternatively, if at least one lethal line for an individual chaperone gene is considered to be a sign of its essential function, the list can be extended to 51 essential chaperones in *Drosophila*.

### Eye-specific knockdown identified several essential chaperones required for rhabdomere biogenesis

The *Drosophila* compound eye has been widely used as an excellent model system to screen for and identify new genes involved in development, eye physiology, and neurodegeneration (Bonini and Fortini 2002). The major advantage of using the *Drosophila* eye as a read out in a screen is that even the subtle morphological defects of eyes can be easily recognized. This allows rapid assessment of phenotype, which can be correlated to cellular defect, disease mechanism, and/or neurodegeneration (Prussing *et al.* 2013; Lu and Vogel 2009). Moreover, the *Drosophila* eye, being a nonvital organ, allows eye-specific expression of disease-related genes to better understand genetic interactions owing to disease onset and progression (Hirth 2010).

**Figure 2:**
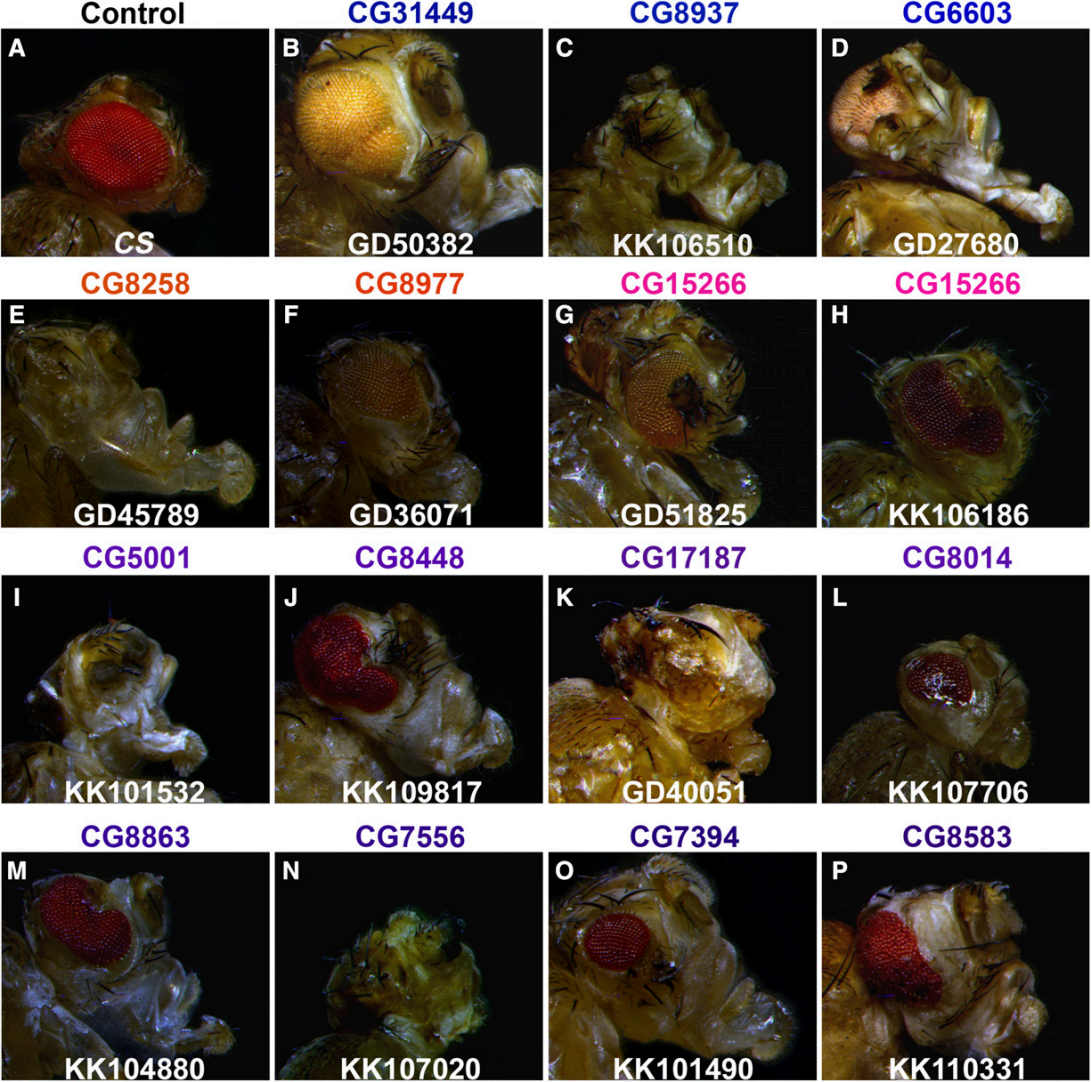
*ey*-Gal4-driven knockdown of candidate essential chaperones alters eye morphology. Photomicrograph of eyes of anesthetized 7-d-old F1 progeny, representing eye-specific knockdown of essential chaperone genes. Canton special (CS) flies crossed with *ey*-Gal4 are used as control (A). Knockdown of essential chaperones belonging to different families, Hsp70 (blue, B–D), Hsp60 (orange, E and F), Prefoldins (magenta, G and H), and Hsp40 (violet, I–P) exhibit a wide range of eye morphology defects.

Hence, in order to further investigate their functional relevance, we knocked-down essential *Drosophila* chaperones in eyes using the *ey*-Gal4 driver. Seventy-six RNAi lines corresponding to 42 essential chaperones were crossed to the *ey*-Gal4 driver. The eyes of anesthetized F1-flies were assessed under a light microscope. Knockdown of several essential chaperones resulted in eye morphological defects suggesting that they are crucial for the development of proper rhabdomeres (Figure 2). The range of eye morphological defects and their penetrance is summarized in Table 2. For three of the chaperones (CG8014, CG7394, and CG8583), only one of the two independent RNAi lines resulted in eye morphological defects. This discrepancy could be due to variability in their knockdown efficiency. In addition, *ey*-Gal4-driven knockdown of 22 lines led to pupal lethality (Table S2) or headless pupae (data not shown). This suggests that *ey*-Gal4 expression is not tightly restricted to the *Drosophila* eyes. Taken together, we identified a set of *Drosophila* chaperones that regulate eye morphogenesis. However, the precise mechanisms by which these individual chaperones regulate eye morphology need to be further addressed.

**Table 2:** Eye-specific knockdown of several *Drosophila* chaperones result in eye morphological defects

Sr. No.	VDRC Line	Gene	Representative Eye Phenotype	% Flies Showing Eye Morphology Defects
Heat shock protein 70 chaperones				
1	GD50382	CG31449	Rough/large eye	100
2	KK106510	CG8937	Eyeless	100
3	GD27680	CG6603	Severely deformed eye	40
Heat shock protein 60 chaperonins				
4	GD45789	CG8258	Severely deformed eye	80
5	GD36071	CG8977	Rough eye	100
Prefoldin				
6	GD51825	CG15266	Ectopic bristles in eyes	50
7	KK106186	CG15266	Deformed eye	100
Heat shock protein40/DnaJ cochaperones				
8	KK101532	CG5001	Eyeless	100
9	KK109817	CG8448	Deformed eye	90
10	GD40051	CG17187	Severely deformed eye	100
11	KK107706	CG8014	Liquid facet-like phenotype	100
12	KK104880	CG8863	Deformed eye	100
13	KK107020	CG7556	Severely deformed eye	90
14	KK101490	CG7394	Small eye, reduced number of ommatidia	100
15	KK110331	CG8583	Rough and deformed eye	100

*ey*-Gal4-driven knockdown identified essential chaperones required for regulating eye morphology and/or rhabdomere development. Knockdown of some of these candidate genes using *ey*-Gal4 also leads to partial pupal lethality. Sr., serial number; No., number; VDRC, Vienna *Drosophila* Resource Center.

### Several essential chaperones regulate NMJ structural development

**Figure 3:**
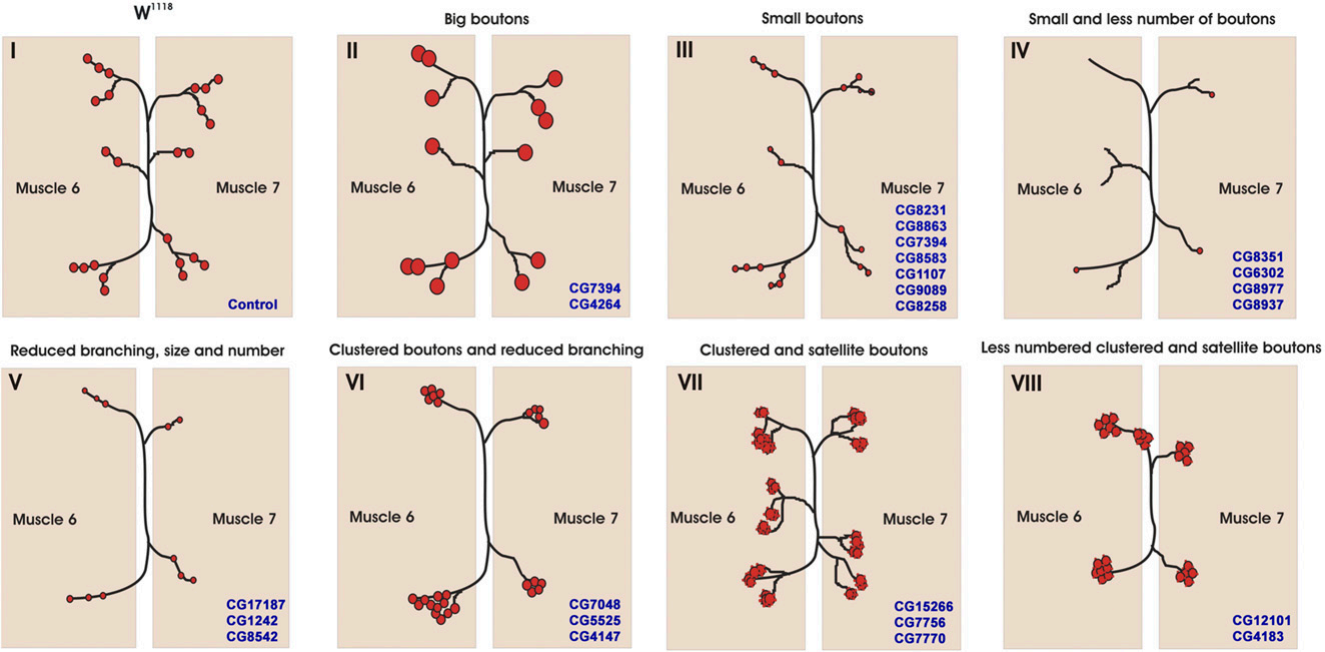
Cartoon representation of the range of neuromuscular junction (NMJ) morphological defects observed due to knockdown of essential chaperones in neurons. Cartoon represents NMJ phenotypic classes upon neuronal depletion of essential chaperones in *Drosophila*. Various NMJ phenotypes were observed and classified into different classes (I–VIII). The NMJ with (I) control, (II) big boutons, (III) small boutons, (IV) small and less number of boutons, (V) reduced branching, size, and number, (VI) clustered boutons and reduced branching, (VII) clustered and satellite boutons, and (VIII) less numbered clustered and satellite boutons are represented.

**Figure 4:**
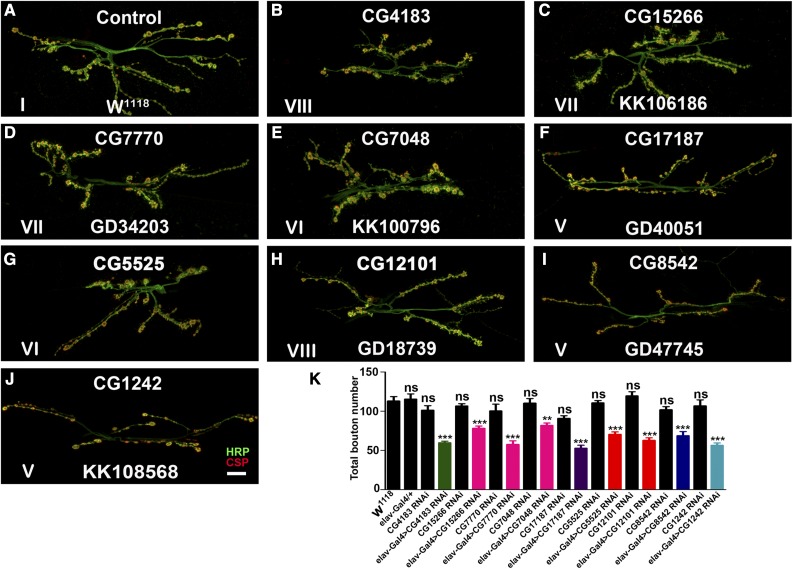
Neuronal depletion of candidate essential chaperones alter NMJ morphology in *Drosophila*. (A–J) Confocal images of NMJ synapses at muscle 6/7 of (A) control, *elav*-Gal4 driven (B) CG4183 RNAi, (C) CG15266 RNAi, (D) CG7770 RNAi, (E) CG7048 RNAi, (F) CG17187 RNAi, (G) CG5525 RNAi, (H) CG12101 RNAi, (I) CG8542 RNAi, and (J) CG1242 RNAi double immunolabeled with a presynaptic marker (CSP, red) and neuronal membrane marker (HRP, green) to reveal the bouton outline at the NMJs. Compared to the control NMJ, *elav*-Gal4-mediated depletion of the above-mentioned essential chaperones showed significantly altered NMJ morphology. The Roman numerals in the image correlate the NMJ morphological defect of each panel with the corresponding phenotypic class depicted in Figure 3. The bouton numbers were determined by manually counting the number of CSP-positive varicosities at the NMJs. Bar in (J) represents 20 µm. (K) Histogram showing average number of boutons at muscle 6/7 of A2 hemisegment in the control, *elav*-Gal4/+, CG4183 RNAi, *elav*-Gal4-driven CG4183 RNAi, CG15266 RNAi, *elav*-Gal4-driven CG15266 RNAi, CG7770 RNAi, *elav*-Gal4-driven CG7770 RNAi, CG7048 RNAi, *elav*-Gal4-driven CG7048 RNAi, CG17187 RNAi, *elav*-Gal4-driven CG17187 RNAi, CG5525 RNAi, *elav*-Gal4-driven CG5525 RNAi, CG12101 RNAi, *elav*-Gal4-driven CG12101 RNAi, CG8542 RNAi, *elav*-Gal4-driven CG8542 RNAi, CG1242 RNAi, and *elav*-Gal4-driven CG1242 RNAi. Histogram in black represents controls including *w*^1118^, *elav*-Gal4/+, and the parental lines. Total number of boutons upon pan-neuronal knockdown of sHSP (green), Prefoldins (magenta), Hsp40 (violet), Hsp60 (orange), Hsp70 (dark blue), and Hsp90 (light blue) have been represented. At least eight NMJ synapses of A2 hemisegments from four larvae of each genotype were used for bouton quantification. ** *P* < 0.001 and *** *P* < 0.0001. Error bars represent SEM (mean ± SEM). Statistical analysis based on one-way ANOVA with *post hoc* Tukey’s test for multiple comparisons. CSP, cysteine string protein; HRP, horseradish peroxidase; Hsp, heat shock protein; NMJ, neuromuscular junction; ns, not significant; RNAi, RNA interference; sHSP, small HSP.

While most of the chaperones are expressed in the nervous system, their role in neuronal development and function remains largely unknown. Hence, to assess the requirement of *Drosophila* chaperones in neurons, we knocked-down essential chaperones using the pan-neuronal driver *elav^C155^*-Gal4 and analyzed the larval NMJ (a specialized synapse formed between motor neurons and muscles) morphology as a read out for possible defects. The NMJs are also among the earliest pathological targets at the onset of several neurological disorders including tauopathies, amyotrophic lateral sclerosis, and spinal muscular atrophy. Neuronal knockdown of these genes identified several chaperones that regulate NMJ structural development in *Drosophila* (Figure 3 and Table S2). We obtained a range of NMJ defects that are summarized in Table S2. All the lines tested for selected genes show more or less similar phenotypes for NMJ morphological defects. The data reported in Table S2 were single blinded, and at least 7 out of 10 NMJs in RNAi knockdown could be identified that differed from control NMJs. However, we have chosen lines that showed 100% penetrance in NMJ morphological defects for representation purpose in Figure 4. Nine of the essential chaperones gave severe NMJ morphological defects (Figure 4). We found that, as compared to the control synapse, neuronal knockdown of these essential chaperones resulted in a significant reduction in the number of boutons (Figure 4K). Taken together, these data suggest that many of the chaperones regulate NMJ morphology in *Drosophila*.

### Microtubule cytoskeleton is disorganized in neuronally depleted essential chaperones

**Figure 5:**
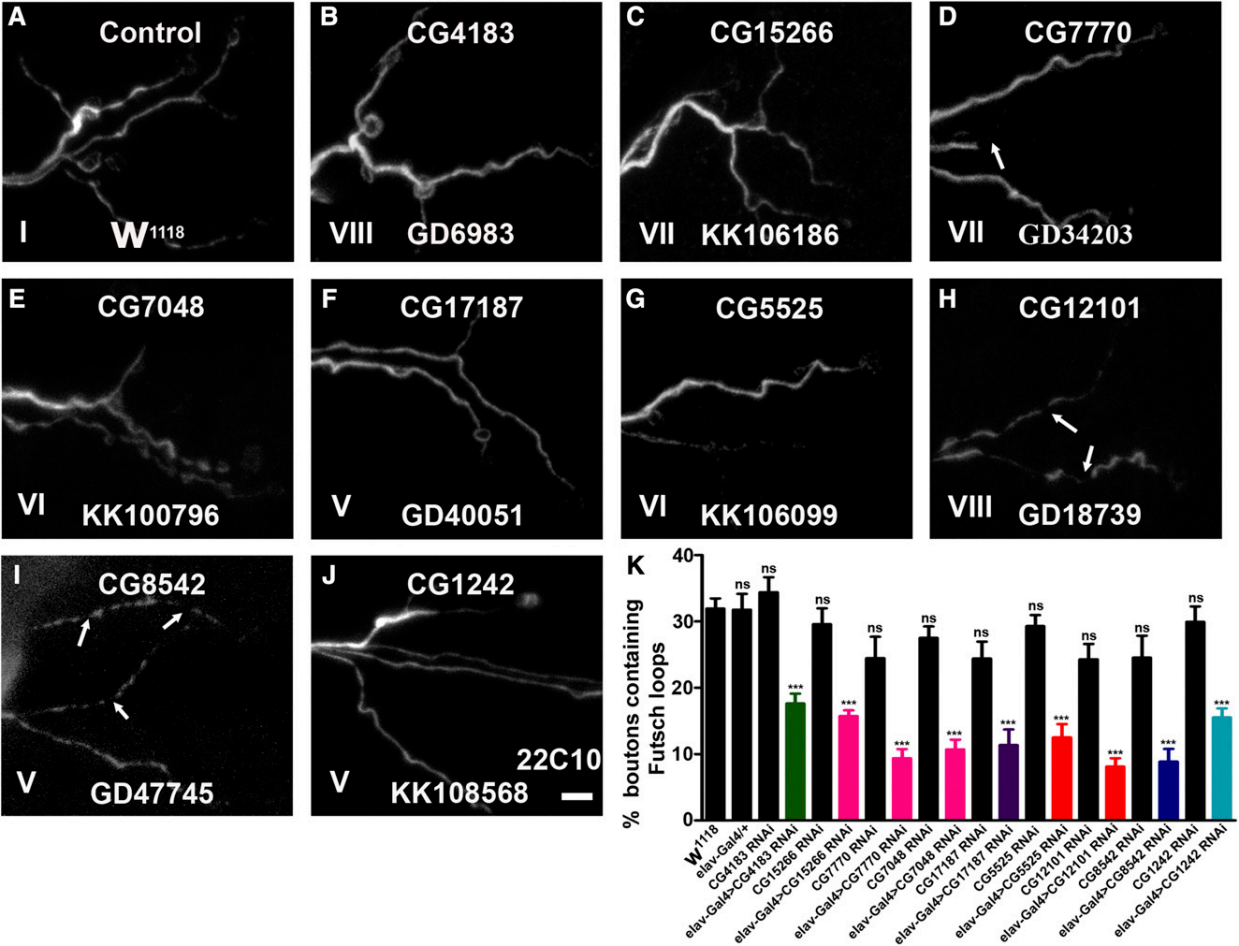
Neuronal knockdown of candidate essential chaperones cause disruption of the presynaptic cytoskeleton. (A–J) Representative images of third instar larval NMJs from muscle 6/7 of A2 hemisegment in (A) control or *elav*-Gal4-driven (B) CG4183 RNAi, (C) CG15266 RNAi, (D) CG7770 RNAi, (E) CG7048 RNAi, (F) CG17187 RNAi, (G) CG5525 RNAi, (H) CG12101 RNAi, (I) CG8542 RNAi, and (J) CG1242 RNAi animals labeled with mAb22C10. Compared to the control NMJ, *elav^C155^*-Gal4-driven knockdown of above essential chaperones shows a significant reduction in the number of Futsch-positive loops and, in some cases, synapses with broken Futsch loops were also seen (marked with arrows). The Roman numerals in the image correlate the NMJ morphological defect of each panel with the corresponding phenotypic class depicted in Figure 3. Bar in (J) represents 4 µm. (K) Histogram showing quantification of the percentage of boutons containing Futsch loops of control, *elav*-Gal4/+, CG4183 RNAi, *elav*-Gal4-driven CG4183 RNAi, CG15266 RNAi, *elav*-Gal4-driven CG15266 RNAi, CG7770 RNAi, *elav*-Gal4-driven CG7770 RNAi, CG7048 RNAi, *elav*-Gal4-driven CG7048 RNAi, CG17187 RNAi, *elav*-Gal4-driven CG17187 RNAi, CG5525 RNAi, *elav*-Gal4-driven CG5525 RNAi, CG12101 RNAi, *elav*-Gal4-driven CG12101 RNAi, CG8542 RNAi, *elav*-Gal4-driven CG8542 RNAi, CG1242 RNAi, and *elav*-Gal4-driven CG1242 RNAi. Histogram in black represents controls including *w*^1118^, *elav*-Gal4/+, and parental lines. Percentage of boutons containing Futsch loops upon pan-neuronal knockdown of sHSP (green), Prefoldins (magenta), Hsp40 (violet), Hsp60 (orange), Hsp70 (dark blue), and Hsp90 (light blue) have been represented. At least eight NMJ synapses from four larvae of each genotype were used for Futsch loop quantification. *** *P* < 0.0001. Error bars represent SEM (mean ± SEM). Statistical analysis based on one-way ANOVA with *post hoc* Tukey’s test for multiple comparisons. Hsp, heat shock protein; NMJ, neuromuscular junction; ns, not significant; RNAi, RNA interference; sHSP, small HSP.

Previous reports suggest that synaptic growth is regulated by microtubule organization and that perturbation of the cytoskeleton affects synaptic growth (Pawson *et al.* 2008). Hence, in order to assess alterations in the microtubule cytoskeleton upon pan-neuronal reduction of essential chaperones, we labeled synapses with an anti-Futsch antibody that labels axonal and nerve terminal cytoskeleton (Roos *et al.* 2000). In the control boutons, the microtubule appeared continuous with periodic loop structures (Figure 5). However, the microtubules in the chaperone-depleted larvae showed significantly reduced microtubule loops (Figure 5K). Interestingly, neuronal reduction of some of the chaperones such as CG12101, CG7770, and CG8542 showed interrupted microtubule assembly at the presynapse. These data support the fact that the identified essential chaperones regulate NMJ structural morphology by regulating the cytoskeletal architecture.

### Neuronal depletion of candidate chaperones result in motor behavior deficits

**Figure 6:**
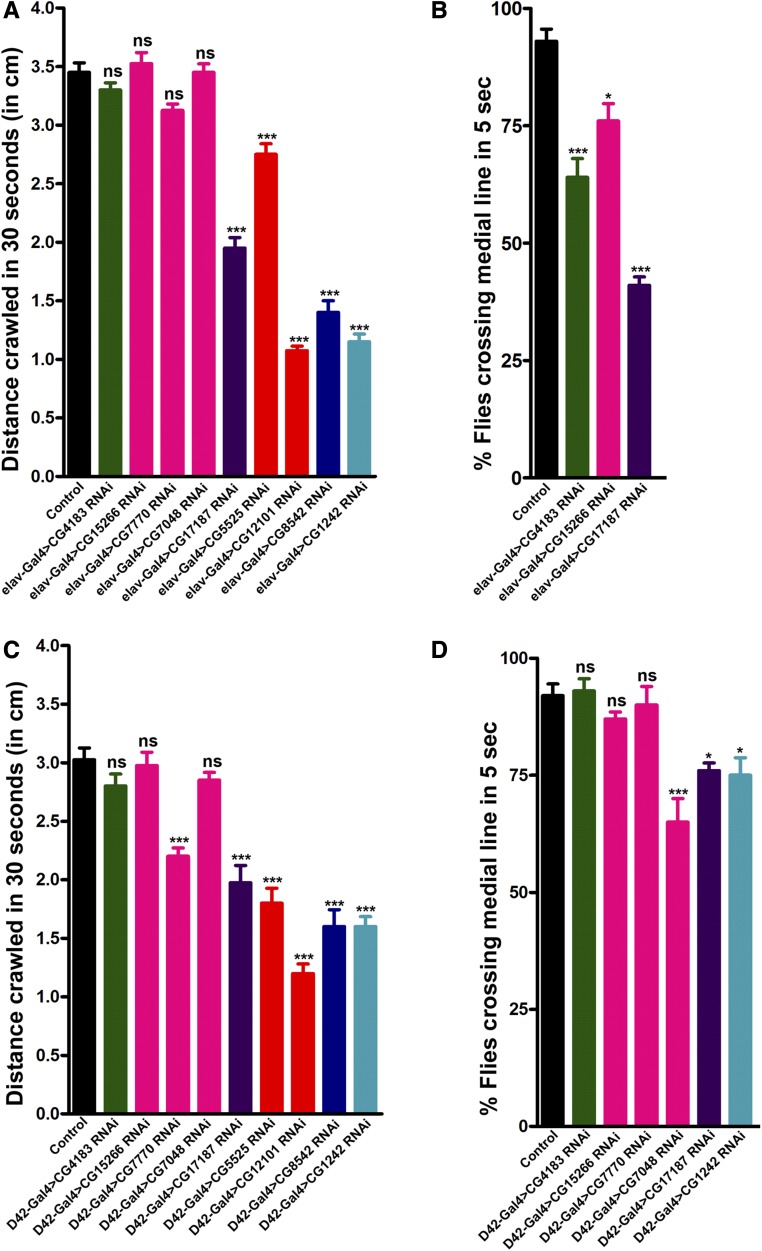
Locomotive behavior affected by pan-neuronal knockdown of essential chaperones. (A) Pan-neuronal downregulation of several essential chaperones affect crawling ability of third instar larvae. Histogram showing average distance crawled in 30 sec by control larvae (3.45 ± 0.08) or larvae with pan-neuronal knockdown of CG4183 (3.3 ± 0.06), CG15266 (3.52 ± 0.09), CG7770 (3.12 ± 0.06), CG7048 (3.45 ± 0.07), CG17187 (1.95 ± 0.09), CG5525 (2.75 ± 0.09), CG12101 (1.08 ± 0.04), CG8542 (1.40 ± 0.10), and CG1242 (1.15 ± 0.06). *n* = 10, *** *P* < 0.0001. Error bars represent SEM (mean ± SEM). Statistical analysis based on one-way ANOVA with *post hoc* Tukey’s test for multiple comparisons. Distance crawled in 30 sec (cm) upon pan-neuronal knockdown of sHSP (green), Prefoldins (magenta), Hsp40 (violet), Hsp60 (orange), Hsp70 (dark blue), and Hsp90 (light blue) have been represented. (B) Pan-neuronal knockdown of essential chaperones affect climbing ability of adult flies. Histogram shows % flies crossing median line in 5 sec in control flies (93.00 ± 2.60) or flies with pan-neuronal knockdown of CG4183 (64.00 ± 4.00), CG15266 (76.00 ± 3.71), and CG17187 (41.00 ± 1.79). *n* = 10, *P < 0.01, ***P < 0.0001. Error bars represent SEM (mean ± SEM). Statistical analysis based on one-way ANOVA with *post hoc* Tukey’s test for multiple comparisons. Percentage of flies crossing the medial line upon pan-neuronal knockdown of sHSP (green), Prefoldins (magenta), and Hsp40 (violet) have been represented. (C) Motor neuron-specific downregulation of several essential chaperones affect crawling ability of third instar larvae. Histogram showing average distance crawled in 30 sec by control larvae (3.03 ± 0.10) or larvae with motor neuron-specific knockdown of CG4183 (2.80 ± 0.10), CG15266 (2.97 ± 0.11), CG7770 (2.20 ± 0.07), CG7048 (2.85 ± 0.07), CG17187 (1.98 ± 0.15), CG5525 (1.80 ± 0.13), CG12101 (1.20 ± 0.08), CG8542 (1.60 ± 0.15), and CG1242 (1.60 ± 0.08). *n* = 10, *** *P* < 0.0001. Error bars represent SEM (mean ± SEM). Statistical analysis based on one-way ANOVA with *post hoc* Tukey’s test for multiple comparisons. Distance crawled in 30 sec (cm) upon motor neuron specific knockdown of sHSP (green), Prefoldins (magenta), Hsp40 (violet), Hsp60 (orange), Hsp70 (dark blue), and Hsp90 (light blue) have been represented. (D) Motor neuron-specific downregulation of several essential chaperones affect climbing ability of adult flies. Histogram shows % flies crossing median line in 5 sec in control flies (92.00 ± 2.49) or flies with motor neuron specific knockdown of CG4183 (93.00 ± 2.60), CG15266 (87.00 ± 1.53), CG7770 (90.00 ± 3.94), CG7048 (65.00 ± 5.00), CG17187 (76.00 ± 1.63), and CG1242 (75.00 ± 3.73). *n* = 10, * *P* < 0.01 and *** *P* < 0.0001. Error bars represent SEM (mean ± SEM). Statistical analysis based on one-way ANOVA with *post hoc* Tukey’s test for multiple comparisons. Percentage of flies crossing the medial line upon motor neuron-specific knockdown of sHSP (green), Prefoldins (magenta), Hsp40 (violet), and Hsp90 (light blue) have been represented. Hsp, heat shock protein; ns, not significant; sHSP, small HSP.

From screening of pan-neuronal knockdown of all essential chaperones, nine candidate genes resulted in severe morphological defects at the NMJ. Aberrant neuronal communication and thus neuronal dysfunction is evident with such severe NMJ morphology defects. Since such defects often lead to compromised locomotor ability (Mudher *et al.* 2004; Mhatre *et al.* 2014), we next examined the effect of pan-neuronal knockdown of these candidate chaperones on locomotive behavior. We analyzed the larval crawling and climbing abilities of adults upon pan-neuronal knockdown of chaperones. Larval crawling ability was significantly reduced upon knockdown of five selected essential chaperones CG1242, CG12101, CG5525, CG8542, and CG17187 (Figure 6A). Compared to the control, we observed significant adult climbing defects upon knockdown of three of these genes (Figure 6B). The climbing ability test could only be performed for three chaperones (CG4183, CG15266, and CG17187) as pan-neuronal knockdown of the rest of the selected chaperones resulted in pupal lethality.

Since pan-neuronal knockdown of most of the selected chaperones resulted in pupal lethality, we used the motor neuron-specific *D42*-Gal4 driver to expand our findings on adult behavior. We performed a larval crawling assay for *D42*-Gal4-driven knockdown of nine selected essential chaperones. Significant defects in crawling ability were observed upon knockdown of six of the nine chaperones (Figure 6C). The adult climbing assay could only be performed for six out of nine selected chaperones, as *D42*-Gal4-driven knockdown of three of the selected chaperones still resulted in pupal lethality (Figure 6D).

The behavioral defect upon neuronal knockdown of several candidate genes is consistent with the NMJ phenotype. It will be interesting to further investigate the functional role of these individual chaperones at the NMJ and to find out whether the behavioral deficit is coupled with their requirement in neurons.

## Discussion

Processes regulating protein turnover and trafficking affect myriad functions in all cells. Given the undeniable role of molecular chaperones in maintaining cellular proteostasis, chaperones are likely to have important roles in neuromorphogenesis, both during development and synaptic plasticity. Consistent with this, several molecular chaperones are reported to play neuroprotective roles, have been linked to various neuropathies (Smith *et al.* 2015), and are considered to be potential therapeutic targets for neurodegenerative disorders (Ebrahimi-Fakhari *et al.* 2013). However, despite their importance in neuronal proteostasis, neurodegeneration, and therapeutics, very little is known about the specific functions of molecular chaperones in neurons. Hence, to elucidate their neuronal functions, we performed an RNAi screen of all canonical chaperones in *Drosophila* and identified several chaperones that play crucial roles in regulating eye morphogenesis and NMJ structural plasticity.

Although ubiquitous, molecular chaperones are best characterized in the unicellular eukaryote *S. cerevisiae* (Gong *et al.* 2009). Thus, using *S. cerevisiae* chaperone sequences as a template for bioinformatics analysis, we identified and classified all *Drosophila* chaperones and established a list of 95 candidates, the most comprehensive list in this organism to date. We followed an unbiased but conservative approach to identify essential chaperones and screened multiple RNAi lines for each chaperone to overcome the limitations of the RNAi approach. As evident from ubiquitous knockdown, there is a greater proportion of essential chaperones in *Drosophila* compared to that in *S. cerevisiae*. For further functional characterization of these essential chaperones, only lethal lines were tested to avoid cases such as inefficient knockdown and lack of remarkable phenotypes, an approach earlier mentioned in a similar screen (Liu *et al.* 2016). Using pan-neuronal and eye-specific Gal4 drivers, lethal lines were screened to identify essential chaperones having possible neuronal functions. Although we suggest that our list of essential chaperones is conservative, we do not rule out the possibility of off-target knockdown of the genes. Genes belonging to the same chaperone family are highly conserved at the nucleotide level. This may lead to the knockdown of multiple members of a chaperone family, in addition to the gene against which the RNAi line was used. Gene-specific knockout of these candidate chaperones would be a more appropriate way to assess their essential function.

We observed varying eye phenotypes upon eye-specific knockdown of almost one-third of essential chaperones. The majority of chaperones exhibiting eye morphological defects were annotated as Hsp40s (also called as J-proteins). Along with their Hsp70 partners, Hsp40s are reported to be involved in maintaining cellular proteostasis by regulating protein folding, protein turnover, and remodeling of macromolecular structures (Hartl *et al.* 2011). For example, HsJ1, a neuronal Hsp40, is required for the sorting of terminally misfolded proteins to the proteasome in *Drosophila* (Westhoff *et al.* 2005). Additionally, J-proteins and Hsp70 have been shown to combat protein aggregation and/or induce apoptosis in cultured neurons (Kobayashi *et al.* 2000), further linking the Hsp70:Hsp40 chaperone machinery with neuronal functions. This is specifically relevant to CG8863 and CG5001, whose yeast counterparts Ydj1 and Sis1 (cytosolic Hsp40s) play important roles in protein folding and clearance of protein aggregates.

Silencing of some of the Hsp70s, Cpn60s, and prefoldin family proteins also resulted in eye morphological defects suggesting important roles in rhabdomere biogenesis. In-depth analysis of the mechanism underlying eye morphological defects due to downregulation of these chaperones will help us better understand the eye-specific function of these essential chaperones. It is likely that *de novo* protein folding, protein turnover, or remodeling of the cytoskeleton may be regulated by these chaperones and that their perturbation causes eye defects. *Eyeless* also expresses in the brain and other parts of the nervous system (Callaerts *et al.* 2001). Consistent with this, our result showed that ∼50% of the essential chaperones resulted in lethality upon *eyeless*-Gal4 driven knockdown, some showing a headless phenotype (for example, CG5525 belonging to the Hsp60 family).

Interestingly, pan-neuronal silencing of many essential chaperones resulted in morphological defects at the third instar larval NMJ. Among these, nine essential chaperones belonging to different families exhibited dramatic changes in NMJ morphology, signifying their importance in synaptic development, growth, and plasticity. Knockdown of these essential chaperones underscores their requirements in possible *de novo* protein folding or their role in mitochondrial function, cytoskeleton organization, neurogenesis, and spliceosome remodeling, all of which may result in altered NMJ morphology. While an imbalance in cellular proteostasis has a marked effect on the NMJ phenotype, one pathway that has been tightly linked with altered NMJ morphology is perturbation of the cytoskeletal architecture (Bodaleo and Gonzalez-Billault 2016). Synaptic morphology is regulated by a neuronal cytoskeleton that maintains the growing end of the synapse. The microtubule-associated neuronal protein Futsch regulates synaptic microtubules, which is essential for synaptic growth at the *Drosophila* NMJ. Futsch loops were shown to be associated with stable synaptic boutons, any deregulation or alteration of which impairs microtubule organization (Roos *et al.* 2000). Molecular chaperones play an important role in cytoskeletal organization and/or cytoskeleton remodeling (Liang and MacRae 1997). Along the same lines, alteration of NMJ morphology was observed upon neuronal depletion of candidate chaperones as a consequence of a perturbed neuronal cytoskeleton. Consistent with the observed cytoskeletal defects, sHsps, prefoldins, and Hsp83 have been shown to either associate with cytoskeletal components or have cytoskeletal remodeling functions (Liang and MacRae 1997). Interestingly, some of these essential chaperones, including CG4183 (sHsp), CG12101 (Hsp60), and CG5525 (Hsp60) have been shown to be microtubule-associated proteins, and CG1242 (Hsp90) is a part of the actin cytoskeleton (Hughes *et al.* 2008; Kiger *et al.* 2003). Defects in NMJ morphology upon neuronal depletion of CG8542 (mortalin) and CG12101 (Hsp60), the mitochondrial Hsp70 and Hsp60, respectively, suggest that mitochondrial protein import and folding is somehow crucial for synaptic development. Perturbation of cellular protein folding and the remodeling of protein aggregates was found to have a severe impact on both eye development and synaptic plasticity. Several selected essential chaperones exhibited defects in locomotive behavior upon pan-neuronal as well as motor neuron-specific knockdown, suggesting their crucial neuron-specific function. One of the surprising candidates that showed significant eye and NMJ phenotypes was the *S. cerevisiae* Cwc23 ortholog (CG17187). RNAi against CG17187 resulted in defective rhabdomere biogenesis and altered NMJ morphology, as well as behavioral deficits. Thus, we surmise that, like Cwc23, CG17187 could also be involved in splicing (Sahi *et al.* 2010). It is likely that defective splicing of multiple genes involved in neuronal functions could lead to pleiotropic defects in eyes and at the NMJ.

Several studies have used the RNAi-mediated knockdown approach to assess the role of chaperones in various cellular contexts. For instance, a genome-wide RNAi screen in *Drosophila* identified Hsp70 as a regulator of intestinal stem cells (Zeng *et al.* 2015). Similarly, an RNAi screen identified Hsp60 and Hsc70 as novel modulators of mitochondrial function (Chen *et al.* 2008); Hsp40 was also identified as a modifier of Huntingtin aggregation (Doumanis *et al.* 2009). In a similar screen, almost 2000 *Drosophila* genes having close human orthologs were screened, which led to the identification of several genes (including chaperones) that affect NMJ growth and maintenance (Valakh *et al.* 2012). We found that out of the 95 chaperones on our list of total *Drosophila* chaperones, 20 were screened by Valakh *et al.* (2012). Thus, one of the reasons why we found a greater number of chaperones affecting NMJ morphology may be the fact that we started with a higher number of candidate chaperones. Moreover, out of nine *Drosophila* chaperones that showed 100% penetrance, six have been tested in a previous screen, and some of these chaperones were also found to be important for NMJ growth and development (Valakh *et al.* 2012). There are some discrepancies in the findings by Valakh *et al.* (2012) and our data. Although we used similar drivers and phenotypic assays, the observed discrepancies are due to different RNAi lines used in these two studies. In addition, Valakh *et al.* (2012) maintained all the lines at 25° for RNAi experiments, whereas we performed all RNAi knockdown experiments at 29°, which could have contributed toward the phenotypic variations between these studies.

To our knowledge, this is the first report of the identification and classification of the *Drosophila* “chaperome,” as well as the only comprehensive screen to identify all essential chaperones. In this screen, we have tried to shortlist the chaperones with neuron-specific function to be further characterized for their mechanism of action. Outcomes of this study and further analysis will provide valuable insights and resources for research on chaperones as possible therapeutic targets for neurodegenerative disease.

## Supplementary Material

Supplemental material is available online at www.g3journal.org/lookup/suppl/doi:10.1534/g3.117.041632/-/DC1.
