# Supplementary material for: RNAi-Mediated Reverse Genetic Screen Identified Drosophila Chaperones Regulating Eye and Neuromuscular Junction Morphology
Source: G3 (Bethesda). 2017 May 8;7(7):2023–38. doi: 10.1534/g3.117.041632 (PMC5499113; doi:10.1534/g3.117.041632)
Supplement: Supplementary file 1 [file 2023TableS1.doc]

| **Sr. No.**  **Table S1. Screening of essential chaperones in *Drosophila melanogaster*** | **Annotation** | **Gene Name** | | **Symbol** | | **VDRC RNAi line** | **Predicted OFF targets** | | | **Viability** | **Essential / Non-essential** |
| --- | --- | --- | --- | --- | --- | --- | --- | --- | --- | --- | --- |
| Small Heat Shock Proteins | | | | | | | | | | | |
| 1 | CG4167 | Heat shock gene 67Ba | | Hsp67Ba | | GD21806 | 22 | | | Viable | **Non-essential** |
|  |  |  |  |  |  | KK104341 | 0 | | | Viable |  |
| 2 | CG4183 | Heat shock protein 26 | | Hsp26 | | GD6983 | 0 | | | Lethal | **Essential** |
|  |  |  |  |  |  | KK100955 | 0 | | | Lethal |  |
| 3 | CG4190 | Heat shock gene 67Bc | | Hsp67Bc | | GD26416 | 0 | | | Viable | **Non-essential** |
|  |  |  |  |  |  | KK103974 | 0 | | | Viable |  |
| 4 | CG4460 | Heat shock protein 22 | | Hsp22 | | GD43632 | 0 | | | Viable | **Non-essential** |
| 5 | CG4461 |  | | CG4461 | | GD40529 | 0 | | | Lethal | **Essential** |
|  |  |  |  |  |  | KK100857 | 1 | | | Lethal |  |
| 6 | CG4463 | Heat shock protein 23 | | Hsp23 | | KK102493 | 2 | | | Lethal | **Essential** |
| 7 | CG4466 | Heat shock protein 27 | | Hsp27 | | GD40530 | 1 | | | Lethal | **Essential** |
|  |  |  |  |  |  | KK101669 | 1 | | | Lethal |  |
| 8 | CG4533 | lethal (2) essential for life | | l(2)efl | | GD40531 | 0 | | | Lethal | **Non-essential** |
|  |  |  |  |  |  | GD40532 | 0 | | | Viable |  |
|  |  |  |  |  |  | KK107305 | 0 | | | Viable |  |
| 9 | CG7409 |  | | CG7409 | | GD40637 | 1 | | | Viable | **Non-essential** |
|  |  |  |  |  |  | KK100517 | 1 | | | Viable |  |
| 10 | CG13133 |  | | CG13133 | | GD52152 | 0 | | | Viable | **Non-essential** |
| 11 | CG14207 |  | | CG14207 | | GD31800 | 0 | | | Viable | **Non-essential** |
|  |  |  |  |  |  | GD31802 | 0 | | | Viable |  |
|  |  |  |  |  |  | GD44831 | 0 | | | Lethal |  |
| Prefoldins | | | | | | | | | | | |
| 1 | CG6302 | Prefoldin 2 | | Pfdn2 | | GD28794 | 0 | | | Lethal | **Essential** |
| 2 | CG6719 | merry-go-round | | Mgr | | GD27727 | 0 | | | Lethal | **Essential** |
| 3 | CG7048 | Prefoldin 5 | | Pfdn5 | | GD29811 | 0 | | | Viable | **Essential** |
|  |  |  |  |  |  | GD29812 | 0 | | | Lethal |  |
|  |  |  |  |  |  | KK100796 | 3 | | | Lethal |  |
| 4 | CG7770 | Prefoldin 6 | | Pfdn6 | | GD34203 | 0 | | | Lethal | **Essential** |
|  |  |  |  |  |  | GD34204 | 0 | | | Lethal |  |
|  |  |  |  |  |  | KK101541 | 0 | | | Lethal |  |
| 5 | CG10635 | Prefoldin 4 | | Pfdn4 | | GD35481 | 3 | | | Lethal | **ND** |
|  |  |  |  |  |  | KK101310 | 2 | | | Viable |  |
| 6 | CG15266 | lethal (2) 35Cc | | l(2)35Cc | | GD51825 | 0 | | | Lethal | **Essential** |
|  |  |  |  |  |  | KK106186 | 0 | | | Lethal |  |
| 7 | CG15676 |  | | CG15676 | | GD19151 | 1 | | | Viable | **Non-essential** |
| Heat Shock Protein 40 | | | | | | | | | | | |
| 1 | CG1107 | | Auxilin | aux | | KK103426 | 1 | Lethal | | | **Essential** |
| 2 | CG1409 | |  | CG1409 | | GD30988 | 0 | Viable | | | **Non-essential** |
|  |  |  |  |  |  | KK110447 | 0 | Viable | | |  |
| 3 | CG1416 | |  | CG1416 | | -- | -- | --- | | | **ND** |
| 4 | CG2239 | | Jdp | Jdp | | GD43044 | 0 | Viable | | | **ND** |
|  |  |  |  |  |  | KK100788 | 0 | Lethal | | |  |
| 5 | CG2790 | |  | CG2790 | | GD20903 | 1 | Viable | | | **Non-essential** |
|  |  |  |  |  |  | KK101619 | 0 | Viable | | |  |
| 6 | CG2887 | |  | CG2887 | | GD33581 | 0 | Viable | | | **Non-Essential** |
|  |  |  |  |  |  | KK106083 | 0 | Viable | | |  |
| 7 | CG2911 | |  | CG2911 | | GD25451 | 0 | Viable | | | **Non-essential** |
|  |  |  |  |  |  | GD25452 | 0 | Viable | | |  |
| 8 | CG3061 | |  | CG3061 | | GD5868 | 1 | Viable | | | **Non-essential** |
| 9 | CG4164 | | Shrivelled | Shv | | GD22996 | 1 | Viable | | | **Non-essential** |
|  |  |  |  |  |  | GD22997 | 1 | Viable | | |  |
|  |  |  |  |  |  | KK108576 | 0 | Viable | | |  |
| 10 | CG4599 | | Tetratricopeptide repeat protein 2 | Tpr2 | | GD26077 | 0 | Viable | | | **Non-essential** |
| 11 | CG5001 | |  | CG5001 | | KK101532 | 3 | Lethal | | | **Essential** |
| 12 | CG5268 | | black pearl | Blp | | GD13794 | 1 | Viable | | | **Non-essential** |
|  |  |  |  |  |  | GD13796 | 1 | Viable | | |  |
| 13 | CG5504 | | lethal (2) tumorous imaginal discs | l(2)tid | | -- |  | --- | | | **ND** |
| 14 | CG6395 | | Cysteine string protein | Csp | | GD34168 | 0 | Viable | | | **Non-essential** |
|  |  |  |  |  |  | KK103201 | 1 | Viable | | |  |
| 15 | CG6693 | |  | CG6693 | | GD27717 | 2 | Viable | | | **Non-Essential** |
|  |  |  |  |  |  | KK109121 | 0 | Viable | | |  |
| 16 | CG7130 | |  | CG7130 | | KK110526 | 0 | Viable | | | **Non-essential** |
| 17 | CG7133 | |  | CG7133 | | GD27876 | 0 | Viable | | | **`Non-essential** |
|  |  |  |  |  |  | GD27878 | 0 | Viable | | |  |
| 18 | CG7387 | |  | CG7387 | | KK101141 | 0 | Viable | | | **Non-essential** |
| 19 | CG7394 | |  | CG7394 | | GD9209 | 0 | Lethal | | | **Essential** |
|  |  |  |  |  |  | GD9210 | 0 | Viable | | |  |
|  |  |  |  |  |  | KK101490 | 0 | Lethal | | |  |
| 20 | CG7556 | |  | CG7556 | | KK107020 | 0 | Lethal | | | **Essential** |
| 21 | CG7872 | |  | CG7872 | | GD9134 | 0 | Viable | | | **Non-essential** |
|  |  |  |  |  |  | KK104410 | 1 | Viable | | |  |
| 22 | CG8014 | | Receptor mediated endocytosis 8 | Rme-8 | | GD22671 | 0 | Lethal | | | **Essential** |
|  |  |  |  |  |  | GD22672 | 0 | Viable | | |  |
|  |  |  |  |  |  | KK107706 | 0 | Lethal | | |  |
| 23 | CG8286 | | P58IPK | P58IPK | | GD14154 | 2 | Viable | | | **Non-essential** |
|  |  |  |  |  |  | KK109649 | 0 | Viable | | |  |
| 24 | CG8448 | | Mrj | Mrj | | KK109817 | 0 | Lethal | | | **Essential** |
| 25 | CG8476 | |  | CG8476 | | KK107172 | 1 | Viable | | | **Non-essential** |
| 26 | CG8531 | |  | CG8531 | | GD24120 | 0 | Viable | | | **ND** |
|  |  |  |  |  |  | KK104088 | 0 | Lethal | | |  |
| 27 | CG8583 | | Secretory 63 | Sec63 | | GD33281 | 1 | Lethal | | | **Essential** |
|  |  |  |  |  |  | GD33282 | 1 | Viable | | |  |
|  |  |  |  |  |  | KK110331 | 0 | Lethal | | |  |
| 28 | CG8863 | | DnaJ-like-2 | Droj2 | | GD23638 | 1 | Lethal | | | **Essential** |
|  |  |  |  |  |  | KK104880 | 1 | Lethal | | |  |
| 29 | CG9089 | | Wurst | Wus | | KK110270 | 0 | Lethal | | | **Essential** |
| 30 | CG9828 | | DnaJ homolog | DnaJ-H | | GD29289 | 0 | Viable | | | **Non-essential** |
| 31 | CG10375 | |  | CG10375 | | GD16039 | 3 | Viable | | | **Non-essential** |
| 32 | CG10565 | |  | CG10565 | | KK105149 | 0 | Viable | | | **Non-essential** |
| 33 | CG10578 | | DnaJ-like-1 | DnaJ-1 | | GD31271 | 0 | Lethal | | | **Essential** |
|  |  |  |  |  |  | KK104618 | 1 | Lethal | | |  |
| 34 | CG11035 | |  | CG11035 | | GD8478 | 0 | Viable | | | **Non-essential** |
|  |  |  |  |  |  | GD8479 | 0 | Viable | | |  |
|  |  |  |  |  |  | GD108566 | 0 | Lethal | | |  |
| 35 | CG12020 | |  | CG12020 | | GD18727 | 0 | Viable | | | **Non-essential** |
|  |  |  |  |  |  | KK104598 | 0 | Viable | | |  |
| 36 | CG14650 | |  | CG14650 | | GD45458 | 3 | Viable | | | **Non-essential** |
|  |  |  |  |  |  | KK107839 | 0 | Viable | | |  |
| 37 | CG17187 | |  | CG17187 | | GD40051 | 1 | Lethal | | | **Essential** |
|  |  |  |  |  |  | KK100297 | 2 | Lethal | | |  |
| 38 | CG30156 | |  | CG30156 | | GD2713 | 0 | Lethal | | | **Essential** |
|  |  |  |  |  |  | GD2714 | 0 | Lethal | | |  |
| 39 | CG32640 | |  | CG32640 | | -- |  | --- | | | **ND** |
| 40 | CG32641 | |  | CG32641 | | -- |  | --- | | | **ND** |
| 41 | CG32727 | |  | CG32727 | | GD48271 | 1 | Viable | | | **Non-essential** |
|  |  |  |  |  |  | KK102503 | 1 | Viable | | |  |
| 42 | CG34246 | | Heat shock protein cognate 20 | Hsc20 | | -- |  | --- | | | **ND** |
| 43 | CG40178 | |  | CG40178 | | KK109162 | 0 | Lethal | | | **Essential** |
|  |  |  |  |  |  | KK110089 | 0 | Lethal | | |  |
| 44 | CG42567 | | DnaJ-like-60 | DnaJ-60 | | GD6655 | 0 | Viable | | | **Non-essential** |
| 45 | CG43322 | |  | CG43322 | | KK110475 | 1 | Viable | | | **Non-essential** |
| Heat Shock Protein 60 | | | | | | | | | | | |
| 1 | CG2830 | | Heat shock protein 60B | | Hsp60B | GD29339 | 0 | Viable | | | **Non-essential** |
|  |  |  |  |  |  | GD29340 | 0 | Viable | | |  |
|  |  |  |  |  |  | KK102092 | 1 | Viable | | |  |
| 2 | CG5374 | | Chaperonin containing TCP1 subunit 1 | | CCT1 | GD34070 | 1 | Viable | | | **Non-essential** |
| 3 | CG5525 | | Chaperonin containing TCP1 subunit 4 | | CCT4 | GD22154 | 0 | Lethal | | | **Essential** |
|  |  |  |  |  |  | GD22155 | 0 | Lethal | | |  |
|  |  |  |  |  |  | KK106099 | 0 | Lethal | | |  |
| 4 | CG6355 | | fab1 kinase | | fab1 | GD27591 | 0 | Viable | | | **Non-essential** |
| 5 | CG7033 | | Chaperonin containing TCP1 subunit 2 | | CCT2 | GD41190 | 3 | Lethal | | | **ND** |
|  |  |  |  |  |  | KK108615 | 2 | Viable | | |  |
| 6 | CG7235 | | Heat shock protein 60C | | Hsp60C | GD47706 | 0 | Viable | | | **Non-essential** |
|  |  |  |  |  |  | GD47708 | 0 | Viable | | |  |
|  |  |  |  |  |  | KK104925 | 0 | Viable | | |  |
| 7 | CG8231 | | Chaperonin containing TCP1 subunit 6 | | CCT6 | GD23751 | 0 | Lethal | | | **Essential** |
|  |  |  |  |  |  | KK109734 | 0 | Lethal | | |  |
| 8 | CG8258 | | Chaperonin containing TCP1 subunit 8 | | CCT8 | GD45789 | 0 | Lethal | | | **Essential** |
|  |  |  |  |  |  | GD45790 | 0 | Viable | | |  |
|  |  |  |  |  |  | KK103905 | 1 | Lethal | | |  |
| 9 | CG8351 | | Chaperonin containing TCP1 subunit 7 | | CCT7 | GD28895 | 0 | Lethal | | | **Essential** |
|  |  |  |  |  |  | KK108585 | 0 | Lethal | | |  |
| 10 | CG8439 | | Chaperonin containing TCP1 subunit 5 | | CCT5 | GD47742 | 1 | Lethal | | | **Essential** |
|  |  |  |  |  |  | KK109505 | 0 | Lethal | | |  |
| 11 | CG8977 | | Chaperonin containing TCP1 subunit 3 | | CCT3 | GD36070 | 0 | Lethal | | | **Essential** |
|  |  |  |  |  |  | GD36071 | 0 | Lethal | | |  |
|  |  |  |  |  |  | KK106093 | 0 | Lethal | | |  |
| 12 | CG12101 | | Heat shock protein 60A | | Hsp60A | GD18738 | 4 | Lethal | | | **Essential** |
|  |  |  |  |  |  | GD18739 | 4 | Lethal | | |  |
|  |  |  |  |  |  | KK100697 | 1 | Lethal | | |  |
| 13 | CG16954 | | Heat shock protein 60D | | Hsp60D | GD19167 | 0 | Viable | | | **Non-essential** |
| Heat Shock Protein 70 | | | | | | | | | | | |
| 1 | CG2918 | |  | CG2918 | | GD18440 | 1 | | Lethal | | **Essential** |
| 2 | CG4147 | | Heat shock 70-kDa protein cognate 3 | Hsc70-3 | | GD14882 | 2 | | Lethal | | **Essential** |
|  |  |  |  |  |  | GD14883 | 2 | | Viable | |  |
|  |  |  |  |  |  | KK101766 | 3 | | Lethal | |  |
| 3 | CG4264 | | Heat shock protein cognate 4 | Hsc70-4 | | GD26465 | 2 | | Lethal | | **Essential** |
|  |  |  |  |  |  | GD50222 | 5 | | Lethal | |  |
|  |  |  |  |  |  | KK101734 | 0 | | Viable | |  |
| 4 | CG5436 | | Heat shock protein 68 | Hsp68 | | GD35007 | 1 | | Lethal | | **Essential** |
|  |  |  |  |  |  | GD47145 | 1 | | Lethal | |  |
|  |  |  |  |  |  | GD47146 | 1 | | Viable | |  |
|  |  |  |  |  |  | KK107356 | 1 | | Lethal | |  |
| 5 | CG5834 | | Hsp70Bbb | Hsp70Bbb | | -- |  | | --- | | **ND** |
| 6 | CG6489 | | Heat-shock-protein-70Bc | Hsp70Bc | | GD26028 | 5 | | Lethal | | **Essential** |
| 7 | CG6603 | | Hsc70Cb | Hsc70Cb | | GD27680 | 2 | | Lethal | | **Essential** |
| 8 | CG7182 | |  | CG7182 | | GD26732 | 2 | | Viable | | **Non-essential** |
|  |  |  |  |  |  | KK101022 | 1 | | Viable | |  |
| 9 | CG7756 | | Heat shock protein cognate 2 | Hsc70-2 | | GD19202 | 8 | | Lethal | | **Essential** |
| 10 | CG8542 | | Heat shock protein cognate 5 | Hsc70-5 | | GD47745 | 2 | | Lethal | | **Essential** |
|  |  |  |  |  |  | KK106236 | 0 | | Lethal | |  |
| 11 | CG8937 | | Heat shock protein cognate 1 | Hsc70-1 | | KK106510 | 1 | | Lethal | | **Essential** |
| 12 | CG18743 | | Heat-shock-protein-70Ab | Hsp70Ab | | GD33207 | 7 | | Viable | | **ND** |
|  |  |  |  |  |  | GD50627 | 6 | | Lethal | |  |
| 13 | CG31359 | | Heat-shock-protein-70Bb | Hsp70Bb | | GD19125 | 5 | | Viable | | **Non-essential** |
|  |  |  |  |  |  | GD36640 | 5 | | Viable | |  |
|  |  |  |  |  |  | GD36641 | 5 | | Viable | |  |
| 14 | CG31366 | | Heat-shock-protein-70Aa | Hsp70Aa | | GD41748 | 5 | | Lethal | | **Essential** |
|  |  |  |  |  |  | GD41749 | 5 | | Lethal | |  |
| 15 | CG31449 | | Heat-shock-protein-70Ba | Hsp70Ba | | GD50381 | 5 | | Lethal | | **Essential** |
|  |  |  |  |  |  | GD50382 | 5 | | Lethal | |  |
| Heat Shock Protein 90 | | | | | | | | | | | |
| 1 | CG1242 | | Heat shock protein 83 | Hsp83 | | GD7716 | 1 | Lethal | | | **Essential** |
|  |  |  |  |  |  | KK108568 | 2 | Lethal | | |  |
| 2 | CG3152 | | Trap1 | Trap1 | | KK108300 | 0 | Viable | | | **Non-essential** |
| 3 | CG5520 | | Glycoprotein 93 | Gp93 | | -- |  | --- | | | **ND** |
| Heat Shock Protein 100 | | | | | | | | | | | |
| 1 | CG4538 | |  | CG4538 | | GD16432 | 0 | Lethal | | | **Essential** |
|  |  |  |  |  |  | GD17250 | 0 | Viable | | |  |
|  |  |  |  |  |  | GD39699 | 0 | Lethal | | |  |

Ubiquitous knockdown of *Drosophila* chaperone identified several essential chaperones. List of all *Drosophila* chaperones along with respective RNAi lines is presented. Each RNAi line was crossed with *actin5C*-Gal4 for ubiquitous knockdown of respective chaperone. F1 progeny were observed for viability or lethality and based upon proportion of lethal lines for each gene essential chaperones were identified. Essential chaperone genes and respective lethal lines are highlighted with yellow colour.
