# Supplementary material for: RNAi-Mediated Reverse Genetic Screen Identified Drosophila Chaperones Regulating Eye and Neuromuscular Junction Morphology
Source: G3 (Bethesda). 2017 May 8;7(7):2023–38. doi: 10.1534/g3.117.041632 (PMC5499113; doi:10.1534/g3.117.041632)
Supplement: Supplementary file 2 [file 2023TableS2.doc]

| **Sr. No.** | **Annotation** | **Gene Name** | **Symbol** | | **VDRC RNAi line** | **Phenotype after Eye specific knockdown** | **Effect upon pan-neuronal knockdown** |
| --- | --- | --- | --- | --- | --- | --- | --- |
| Small Heat Shock Proteins  **Table S2. Effects of eye-specific and pan-neuronal knockdown of essential chaperones in *Drosophila melanogaster*** | | | | | | | |
| 1 | CG4183 | Heat shock protein 26 | Hsp26 | | GD6983 | Normal | Clustered, less number and satellite boutons |
|  |  |  |  |  | KK100955 | Normal | Small boutons |
| 2 | CG4461 |  | CG4461 | | GD40529 | Normal | Normal |
|  |  |  |  |  | KK100857 | Normal | Normal |
| 3 | CG4463 | Heat shock protein 23 | Hsp23 | | KK102493 | Normal | Normal |
| 4 | CG4466 | Heat shock protein 27 | Hsp27 | | GD40530 | Normal | Normal |
|  |  |  |  |  | KK101669 | Normal | Normal |
| Prefoldin family proteins | | | | | | | |
| **1** | CG6302 | Prefoldin 2 | Pfdn2 | | GD28794 | Normal | Less number of boutons |
| **2** | CG6719 | merry-go-round | Mgr | | GD27727 | Normal | Normal |
| **3** | CG7048 | Prefoldin 5 | Pfdn5 | | GD29812 | Normal | Reduced branching and severely clustered boutons |
|  |  |  |  |  | KK100796 | Normal | Boutons with reduced number and size |
| **4** | CG7770 | Prefoldin 6 | Pfdn6 | | GD34203 | Pupal lethal | Clustered and satellite boutons |
|  |  |  |  |  | GD34204 | Pupal lethal | Boutons with reduced number and size |
|  |  |  |  |  | KK101541 | Pupal lethal | Normal |
| **5** | CG15266 | lethal (2) 35Cc | l(2)35Cc | | GD51825 | Ectopic bristle in eyes | Small boutons |
|  |  |  |  |  | KK106186 | Deformed eye | Clustered and satellite boutons |
| Heat Shock Protein 40 | | | | | | | |
| **1** | CG1107 | auxilin | Aux | | KK103426 | Pupal lethal | Small boutons |
| **2** | CG5001 |  | CG5001 | | KK101532 | Severely deformed eye | Normal |
| **3** | CG7394 |  | CG7394 | | GD9209 | Normal | Small boutons |
|  |  |  |  |  | KK101490 | Small eye with reduced number of ommatidia | Clustered big boutons |
| **4** | CG7556 |  | CG7556 | | KK107020 | Severely deformed eye | Normal |
| **5** | CG8014 | Receptor mediated endocytosis 8 | Rme-8 | | GD22671 | Normal | Normal |
|  |  |  |  |  | KK107706 | Liquid facet like phenotype | Normal |
| **6** | CG8448 | mrj | Mrj | | KK109817 | Deformed eye | Normal |
| **7** | CG8583 | Secretory 63 | Sec63 | | GD33281 | Normal | Small boutons |
|  |  |  |  |  | KK110331 | Rough and deformed eye | Normal |
| **8** | CG8863 | DnaJ-like-2 | Droj2 | | GD23638 | ND | ND |
|  |  |  |  |  | KK104880 | Deformed eye | Small boutons |
| **9** | CG9089 | wurst | Wus | | KK110270 | Pupal lethal | Small boutons |
| **10** | CG10578 | DnaJ-like-1 | DnaJ-1 | | GD31271 | Normal | Several Ghost buttons |
|  |  |  |  |  | KK104618 | Normal | ND |
| **11** | CG17187 |  | CG17187 | | GD40051 | Severely deformed eye | Reduced branching and boutons with reduced number and size |
|  |  |  |  |  | KK100297 | Pupal lethal | ND |
| **12** | CG30156 |  | CG30156 | | GD2713 | Normal | Normal |
|  |  |  |  |  | GD2714 | Normal | Normal |
| **13** | CG40178 |  | CG40178 | | KK109162 | Normal | Normal |
|  |  |  |  |  | KK110089 | Normal | Normal |
| Heat Shock Protein 60 | | | | | | | |
| **1** | CG5525 | Chaperonin containing TCP1 subunit 4 | CCT4 | | GD22154 | Pupal lethal | Boutons with reduced number and size |
|  |  |  |  |  | GD22155 | ND | Small boutons |
|  |  |  |  |  | KK106099 | Pupal lethal | Clustered and less number of boutons |
| **2** | CG8231 | Chaperonin containing TCP1 subunit 6 | CCT6 | | GD23751 | ND | Small boutons |
|  |  |  |  |  | KK109734 | Pupal lethal | Clustered boutons |
| **3** | CG8258 | Chaperonin containing TCP1 subunit 8 | CCT8 | | GD45789 | Severely deformed eye | Normal |
|  |  |  |  |  | KK103905 | Pupal lethal | Small boutons |
| **4** | CG8351 | Chaperonin containing TCP1 subunit 7 | CCT7 | | GD28895 | Pupal lethal | Less number of boutons |
|  |  |  |  |  | KK108585 | Pupal lethal | ND |
| **5** | CG8439 | Chaperonin containing TCP1 subunit 5 | CCT5 | | GD47742 | Normal | ND |
|  |  |  |  |  | KK109505 | Pupal lethal | ND |
| **6** | CG8977 | Chaperonin containing TCP1 subunit 3 | CCT3 | | GD36070 | Pupal lethal | Boutons with reduced number and size |
|  |  |  |  |  | GD36071 | Reduced number of ommatidia | Boutons with reduced number and size |
|  |  |  |  |  | KK106093 | Pupal lethal | Normal |
| **7** | CG12101 | Heat shock protein 60A | Hsp60A | | GD18738 | Normal | Less number of boutons |
|  |  |  |  |  | GD18739 | Pupal lethal | Reduced branching and less number, smaller size and satellite boutons |
|  |  |  |  |  | KK100697 | Pupal lethal | Small elongated boutons |
| Heat Shock Protein 70 | | | | | | | |
| **1** | CG2918 |  | | CG2918 | GD18440 | Normal | Normal |
| **2** | CG4147 | Heat shock 70-kDa protein cognate 3 | | Hsc70-3 | GD14882 | Pupal lethal | Normal |
|  |  |  |  |  | KK101766 | Pupal lethal | Small, clustered boutons |
| **3** | CG4264 | Heat shock protein cognate 4 | | Hsc70-4 | GD26465 | ND | Big boutons |
|  |  |  |  |  | GD50222 | Normal | Normal |
| **4** | CG5436 | Heat shock protein 68 | | Hsp68 | GD35007 | Normal | ND |
|  |  |  |  |  | GD47145 | ND | Normal |
|  |  |  |  |  | KK107356 | Normal | Normal |
| **5** | CG6489 | Heat-shock-protein-70Bc | | Hsp70Bc | GD26028 | ND | Normal |
| **6** | CG6603 | Hsc70Cb | | Hsc70Cb | GD27680 | Deformed eye | Normal |
| **7** | CG7756 | Heat shock protein cognate 2 | | Hsc70-2 | GD19202 | Pupal lethal | Several satellite boutons, reminiscent of adaptive mutation |
| **8** | CG8542 | Heat shock protein cognate 5 | | Hsc70-5 | GD47745 | Pupal lethal | Reduced branching, boutons with severely reduced number and size |
|  |  |  |  |  | KK106236 | Normal | Small boutons |
| **9** | CG8937 | Heat shock protein cognate 1 | | Hsc70-1 | KK106510 | Severely deformed eye | Less number of boutons |
| **10** | CG31366 | Heat-shock-protein-70Aa | | Hsp70Aa | GD41748 | Pupal lethal | Extensive branching |
|  |  |  |  |  | GD41749 | ND | ND |
| **11** | CG31449 | Heat-shock-protein-70Ba | | Hsp70Ba | GD50381 | Pupal lethal | ND |
|  |  |  |  |  | GD50382 | Rough and large eye | ND |
| Heat Shock Protein 90 | | | | | | | |
| **1** | CG1242 | Heat shock protein 83 | | Hsp83 | GD7716 | Pupal lethal | Reduced boutons |
|  |  |  |  |  | KK108568 | Pupal lethal | Reduced branching and less number of boutons |
| Heat Shock Protein 100 | | | | | | | |
| **1** | CG4538 |  | | CG4538 | GD16432 | Normal | Normal |
|  |  |  |  |  | GD39699 | Normal | Normal |

Eye specific and pan-neuronal knockdown of all essential *Drosophila* chaperones identified several candidates affecting eye development and NMJ morphology. List of all essential *Drosophila* chaperones along with respective lethal RNAi lines is presented. Each RNAi line was crossed with *ey*-Gal4 and *elav*-Gal4. The representative eye phenotypes and NMJ morphological defects are highlighted with yellow colour.
