# Supplementary material for: RNAi-Mediated Reverse Genetic Screen Identified Drosophila Chaperones Regulating Eye and Neuromuscular Junction Morphology
Source: G3 (Bethesda). 2017 May 8;7(7):2023–38. doi: 10.1534/g3.117.041632 (PMC5499113; doi:10.1534/g3.117.041632)
Supplement: Supplementary file 3 [file 2023TableS3.docx]

**Table S3. Genotypes , NMJ morphology and Futsch quantification at muscle 6/7 in abdominal segement A2. N represents the number of NMJ used for quantification.**

| **Genotype** | **Total**  **bouton number** | **% boutons containing**  **Futsch loops** |
| --- | --- | --- |
| Control | 113.0 ± 5.57 N=8 | 31.95 ± 1.51 N=8 |
| *elav*-Gal4/+ | 115.4 ± 6.66 N=8 | 31.76 ± 2.40 N=8 |
| CG4183 RNAi | 101.3 ± 5.83 N=8 | 34.41 ± 2.25 N=8 |
| *elav*-Gal4>CG4183 RNAi | 59.75 ± 2.00 N=8 | 17.63 ± 1.49 N=8 |
| CG15266 RNAi | 106.6 ± 2.87 N=8 | 29.58 ± 2.41 N=8 |
| *elav*-Gal4>CG15266 RNAi | 78.25 ± 2.86 N=8 | 15.75 ± 0.86 N=8 |
| CG7770 RNAi | 100.4 ± 8.57 N=8 | 24.48 ± 3.21 N=8 |
| *elav*-Gal4>CG7770 RNAi | 57.63 ± 4.60 N=8 | 9.39 ± 1.36 N=8 |
| CG7048 RNAi | 110.3 ± 5.86 N=8 | 27.53 ± 1.72 N=8 |
| *elav*-Gal4>CG7048 RNAi | 81.88 ± 3.09 N=8 | 10.73 ± 1.46 N=8 |
| CG17187 RNAi | 90.88 ± 3.31 N=8 | 24.41 ± 2.55 N=8 |
| *elav*-Gal4>CG17187 RNAi | 52.88 ± 3.77 N=8 | 11.37 ± 2.34 N=8 |
| CG5525 RNAi | 110.6 ± 2.92 N=8 | 29.30 ± 1.67 N=8 |
| *elav*-Gal4>CG5525 RNAi | 70.50 ± 3.15 N=8 | 12.53 ± 2.01 N=8 |
| CG12101 RNAi | 119.5 ± 5.25 N=8 | 24.30 ± 2.31 N=8 |
| *elav*-Gal4>CG12101 RNAi | 62.88 ± 3.16 N=8 | 8.13 ± 1.25 N=8 |
| CG8542 RNAi | 101.9 ± 3.92 N=8 | 24.54 ± 3.31 N=8 |
| *elav*-Gal4>CG8542 RNAi | 68.75 ± 5.28 N=8 | 8.87 ± 1.90 N=8 |
| CG1242 RNAi | 106.8 ± 7.678 N=8 | 29.94 ± 2.33 N=8 |
| *elav*-Gal4>CG1242 RNAi | 56.50 ± 3.071 N=8 | 15.54 ± 1.34 N=8 |
